# Identification of unusual oxysterols and bile acids with 7-oxo or 3β,5α,6β-trihydroxy functions in human plasma by charge-tagging mass spectrometry with multistage fragmentation[S]

**DOI:** 10.1194/jlr.D083246

**Published:** 2018-04-06

**Authors:** William J. Griffiths, Ian Gilmore, Eylan Yutuc, Jonas Abdel-Khalik, Peter J. Crick, Thomas Hearn, Alison Dickson, Brian W. Bigger, Teresa Hoi-Yee Wu, Anu Goenka, Arunabha Ghosh, Simon A. Jones, Yuqin Wang

**Affiliations:** Swansea University Medical School,* Swansea SA2 8PP, Wales, United Kingdom; Stem Cell and Neurotherapies, Division of Cell Matrix Biology and Regenerative Medicine,† University of Manchester, Manchester M13 9PT, United Kingdom; Manchester Centre for Genomic Medicine, St. Mary’s Hospital, Central Manchester Foundation Trust,§ University of Manchester, Manchester M13 9WL, United Kingdom

**Keywords:** sterols, cholesterol/metabolism, Niemann-Pick type C, oxidized lipids, tandem mass spectrometry

## Abstract

7-Oxocholesterol (7-OC), 5,6-epoxycholesterol (5,6-EC), and its hydrolysis product cholestane-3β,5α,6β-triol (3β,5α,6β-triol) are normally minor oxysterols in human samples; however, in disease, their levels may be greatly elevated. This is the case in plasma from patients suffering from some lysosomal storage disorders, e.g., Niemann-Pick disease type C, or the inborn errors of sterol metabolism, e.g., Smith-Lemli-Opitz syndrome and cerebrotendinous xanthomatosis. A complication in the analysis of 7-OC and 5,6-EC is that they can also be formed ex vivo from cholesterol during sample handling in air, causing confusion with molecules formed in vivo. When formed endogenously, 7-OC, 5,6-EC, and 3β,5α,6β-triol can be converted to bile acids. Here, we describe methodology based on chemical derivatization and LC/MS with multistage fragmentation (MS^n^) to identify the necessary intermediates in the conversion of 7-OC to 3β-hydroxy-7-oxochol-5-enoic acid and 5,6-EC and 3β,5α,6β-triol to 3β,5α,6β-trihydroxycholanoic acid. Identification of intermediate metabolites is facilitated by their unusual MS^n^ fragmentation patterns. Semiquantitative measurements are possible, but absolute values await the synthesis of isotope-labeled standards.

Until recently, 7-oxocholesterol (7-OC), 5,6-epoxycholesterol (5,6-EC), and its hydrolysis product cholestane-3β,5α,6β-triol (3β,5α,6β-triol) were regarded by many as artifacts generated by sample handling of cholesterol-rich material in air (1–4). For a list of sterol abbreviations, see supplemental Table S1. That view has changed with the discovery that 7-OC can be generated enzymatically from the cholesterol precursor 7-dehydrocholesterol (7-DHC) by cytochrome P450 (CYP) 7A1 (5) and is abundant in plasma of patients with Smith-Lemli-Opitz Syndrome (SLOS), where levels of 7-DHC are high, and cerebrotendinous xanthomatosis, where CYP7A1 is highly expressed (6, 7). Furthermore, in patients with lysosomal storage disorders Niemann-Pick (NP) disease types C and B and lysosomal acid lipase deficiency, 3β,5α,6β-triol is elevated in plasma, as is 7-OC, despite apparently normal levels of 7-DHC (8–13). For an up-to-date review, see ref. 14. Importantly, recent reports by Clayton and colleagues in London (15) and Ory and colleagues in St Louis (16) have documented the presence of the unusual bile acid 3β,5α,6β-trihydroxycholanoic acid (3β,5α,6β-triHBA) in the plasma of NPC patients, while Alvelius et al. and Maekawa et al. have reported the presence of the sulfuric acid and glycine conjugates of 3β-hydroxy-7-oxochol-5-enoic acid (3βH,7O-Δ^5^-BA) in urine and plasma of NPC patients (17, 18). The observation of these unusual bile acids associated with NPC and of 3βH,7O-Δ^5^-BA with other disorders, e.g., SLOS (19–21), strongly suggests that their precursors 7-OC and 5,6-EC are formed in vivo and are not (only) ex vivo artifacts generated through sample handling in air. Both 7-OC and 5,6-EC are dietary oxysterols (22, 23), while 5,6-EC may also be formed by environmental ozone in lung (24), representing alternative sources of these molecules in healthy individuals. In fact, Lyons et al. showed that 7-OC was rapidly metabolized by the liver in rats and excreted into the intestine mainly as aqueous soluble metabolites, presumably bile acids (22). Pulfer and Murphy showed that 5,6-EC was the major cholesterol-derived product formed in the reaction of ozone with lung surfactant and that 3β,5α,6β-triol, and more abundant levels of an unexpected metabolite, 3β,5α-dihydroxycholestan-6-one, were formed from 5,6-EC by lung epithelial cells (24).

To investigate how 7-OC is metabolized in vivo into 3βH,7O-Δ^5^-BA and 5,6-EC and 3β,5α,6β-triol into 3β,5α,6β-triHBA, we have optimized a charge-tagging methodology to specifically identify 7-oxo-containing sterols and sterols with a 3β,5α,6β-triol function using chemical derivatization and LC/MS with multistage fragmentation (MS^n^). The resultant method is described below.

## MATERIALS AND METHODS

### Human samples

Plasma was from patients diagnosed with lysosomal storage disorders, their siblings, or parents. All participants or their parents provided written informed consent in accordance with the Declaration of Helsinki, and the study was conducted with institutional review board approval (REC08/H1010/63). National Institute of Standards and Technology (NIST) standard reference material (SRM1950, Gaithersburg, MD), a pooled plasma sample representative of the US population (25), was used as a reference.

### Materials

Oxysterols and C_27_ bile acids were from Avanti Polar Lipids Inc. (Alabaster, AL); C_24_ bile acids were a kind gift from Professor Jan Sjövall (Karolinska Institutet, Stockholm, Sweden); 3β,5α,6β-triHBA was a kind gift from Professor Douglas F. Covey (Washington University School of Medicine). See supplemental Table S1 for a list of oxysterols and bile acid with their common and systematic names, abbreviations, LipidMaps ID, and suppliers. Cholesterol oxidase (ChOx) enzyme from *Streptomyces* sp. was from Sigma-Aldrich Ltd. (Dorset, UK), [^2^H_0_]Girard P ([^2^H_0_]GP) reagent was from TCI Europe (Zwijndrecht, Belgium), and [^2^H_5_]GP was synthesized as described in ref. 26. Reversed-phase Certified Sep-Pak C_18_ (200 mg) and Oasis HLB (60 mg) solid phase extraction (SPE) columns were from Waters Ltd (Elstree, Herts, UK).

### Sample preparation

The sample preparation protocol is described in detail in ref. 27 and only differs here by the addition of additional deuterium-labeled standards. In brief, plasma (100 µl) was added to absolute ethanol (1.05 ml) containing deuterated internal standards, including [25,26,26,26,27,27,27-^2^H_7_]7-OC ([^2^H_7_]7-OC), [25,26,26,26,27,27,27-^2^H_7_]5α,6-EC ([^2^H_7_]5α,6-EC), [25,26,26,26,27,27,27-^2^H_7_]­3β,5α,6β-triol ([^2^H_7_]3β,5α,6β-triol), [25,26,26,26,27,27,27-^2^H_7_]­7α-hydroxycholesterol ([^2^H_7_]7α-HC), [25,26,26,26,27,27,27-^2^H_7_]24R/S-hydroxycholesterol ([^2^H_7_]24R/S-HC), [26,26,26,27,27,27-^2^H_6_]7α,25-dihydroxycholesterol ([^2^H_6_]7α,25-diHC), [25,26,26,26,27,27,27-^2^H_7_]22R-hydroxycholest-4-en-3-one ([^2^H_7_]22R-HCO), [26,26,26,27,27,27-^2^H_6_]25-hydroxyvitamin D_3_, and [25,26,26,26,27,27,27-^2^H_7_]cholesterol ([^2^H_7_]C). The solution was diluted to 70% ethanol with 0.35 ml of water, sonicated, and centrifuged to remove precipitated matter. To separate bile acids and oxysterols from cholesterol and similarly hydrophobic sterols, the sample solution was applied to a 200 mg Sep-Pack C_18_ column; cholesterol was absorbed while oxysterols and bile acids eluted in the flow-through and column wash (SPE1-Fr1, 7 ml 70% ethanol). After a further column wash (SPE1-Fr2, 4 ml 70% ethanol), cholesterol and sterols of similar hydrophobicity were then eluted in a separate fraction with absolute ethanol (SPE1-Fr3, 2 ml). The oxysterol/bile acid (SPE1-Fr1) and cholesterol-rich (SPE1-Fr3) fractions were then each divided into two equal aliquots (A) and (B) and lyophilized. After reconstitution in propan-2-ol (100 µl), ChOx (0.26 units) in 50 mM phosphate buffer (1 ml), pH 7, was added to subfractions (A). After 1 h at 37°C, the reaction was quenched with methanol (2 ml). Subfractions (B) were treated in an identical manner but in the absence of ChOx. Glacial acetic acid (150 µl) was added to each subfraction, followed by [^2^H_5_]GP as the bromide salt (190 mg) to subfractions (A) and [^2^H_0_]GP as the chloride salt (150 mg) to subfractions (B). The derivatization reactions were left to proceed overnight in the dark at room temperature. Excess derivatization reagent was removed by SPE using a recycling method. Each subfraction (now 3.25 ml, 69% organic) was applied to a 60 mg Oasis HLB column and washed with 70% methanol (1 ml) and 35% methanol (1 ml). The combined effluent was diluted to 35% methanol and recycled through the column. This was repeated with dilution to 17.5% methanol and a further recycling, and the column was finally washed with 10% methanol (6 ml). At this point, all oxysterols/bile acids, or more hydrophobic sterols, were absorbed on the column while unreacted GP reagent elute to waste. Oxysterols/bile acids were then eluted with 100% methanol (2 ml, SPE2-Fr1+2), while more hydrophobic sterols with 3 ml of 100% methanol (SPE2-Fr1+2+3). Just prior to LC/MS(MS^n^) analysis, equal volumes of subfractions (A) and (B) were combined and diluted to 60% methanol ready for injection. This allowed the simultaneous analysis of subfractions A and B.

### LC/MS(MS^n^)

Analysis was performed on an Orbitrap Elite (Thermo Fisher Scientific, Hemel Hempstead, UK) exploiting electrospray ionization. Chromatographic separation was achieved with a reversed-phase Hypersil Gold column (1.9 µm particle size, 50 × 2.1 mm, Thermo Fisher) using an Ultimate 3000 LC system (now Thermo Fisher Scientific) with the mobile phase and gradient described in refs. 26 and 27. To separate some closely eluting oxysterols/bile acids, the usual 17 min gradient was extended to 37 min. The mobile phase composition was initially at 80% A (33.3% methanol, 16.7% acetonitrile, and 0.1% formic acid) and 20% B (63.3% methanol, 31.7% acetonitrile, and 0.1% formic acid) for 10 min, changed to 50% A, 50% B over the next 10 min, maintained at this composition for 6 min, and then changed to 20% A, 80% B over the next 3 min. The mobile phase composition was held at 20% A, 80% B for a further 3 min before returning to 80% A, 20% B in 0.1 min and reconditioning the column for a further 4.9 min.

For each injection, five scan events were performed: one high-resolution scan (120,000 full-width at half maximum height definition at *m/z* 400) in the Orbitrap analyzer in parallel to four MS^3^ scan events in the linear ion-trap. Quantification was by isotope dilution or by using isotope-labeled structural analogs.

## RESULTS

### 7-Oxo containing sterols

**Fig. 1.**
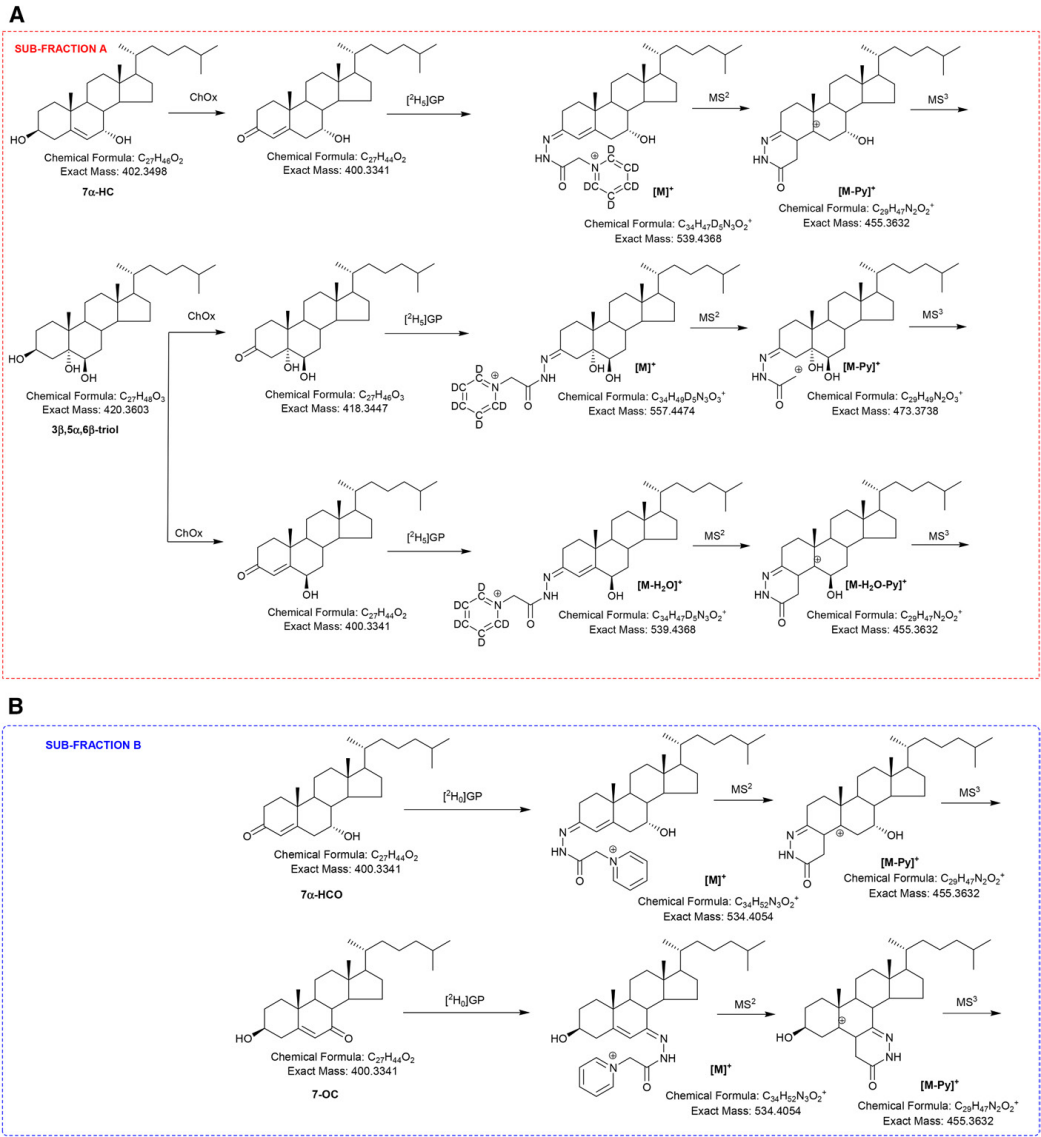
A: Oxidation of 3β-hydroxysterols with ChOx, then derivatization with [^2^H_5_]GP and MS^n^ fragmentation. B: Derivatization of oxosterols with [^2^H_0_]GP and MS^n^ fragmentation.

**Fig. 2.**
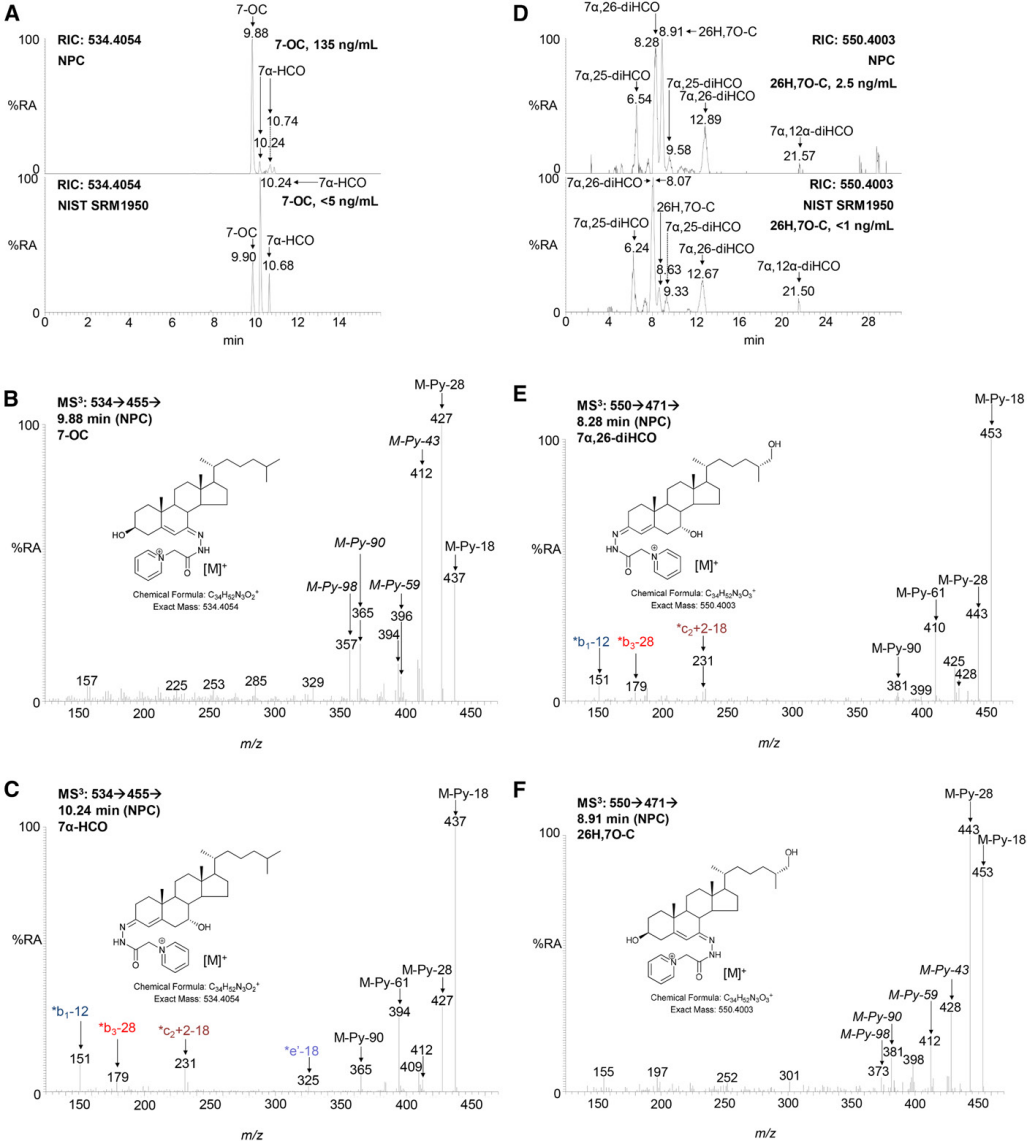
Chromatographic separation and MS^3^ ([M]^+^→[M-Py]^+^→) spectra of 7-OC, 7α-HCO, 26H,7O-C, and 7α,26-diHCO from an NPC plasma sample derivatized with [^2^H_0_]GP reagent. A: RICs (*m/*z 534.4054 ± 5 ppm) demonstrating the separation of 7-OC from 7α-HCO in a plasma sample from an NPC (upper) patient and in the NIST reference material (lower). MS^3^ spectra of 7-OC (B) and 7α-HCO (C) from the NPC plasma sample. D: RIC (550.4003 ± 5 ppm) demonstrating chromatographic separation of 26H,7O-C from 7α,26-diHCO and other isomers in the NPC (upper) and NIST (lower) plasma samples using the 37 min chromatographic gradient. The chromatograms in D were recorded on different days, resulting in an offset in retention time of 0.2–0.3 min in the earlier peaks. MS^3^ spectra of 7α,26-diHCO (E) and 26H,7O-C (F) from the NPC sample. Measured concentrations of 7-OC and 26H,7-OC are given in the right-hand corners of the chromatograms A and D, respectively. Note that 7α-HCO, 7α,25-diHCO, and 7α,26-diHCO give twin peaks corresponding to *syn* and *anti* conformers of the derivative. MS^3^ spectra of authentic standards of [^2^H_7_]7-OC, 7-OC, 7α-HCO, 26H,7O-C, 7α,26-diHCO, 7α,25-diHCO, and 7α,24S-diHCO can be found in supplemental Fig. S2A–G. %RA, % relative abundance.

**Fig. 3.**
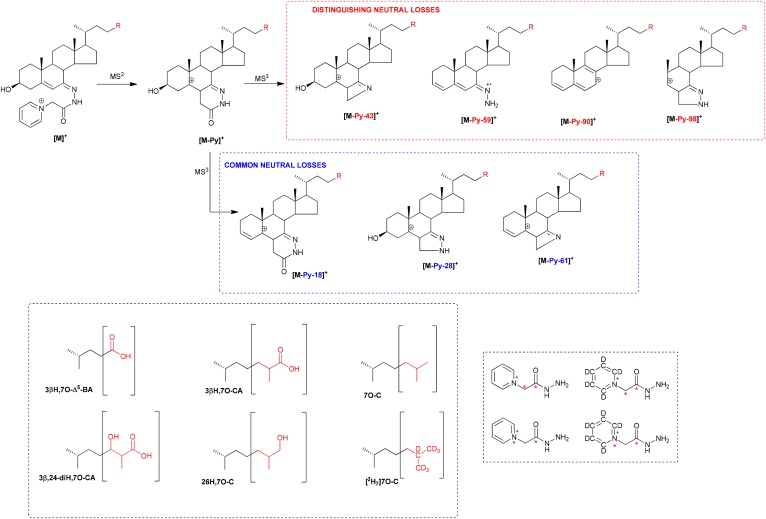
Patterns of MS^3^ ([M]^+^→[M-Py]^+^→) neutral losses, which distinguish between, or are common to, [^2^H_0_]GP derivatized 7-oxo-5-ene and 3-oxo-4-ene sterols. Structures of R groups are shown within brackets in the lower left-hand box. Isotope-labeled [^13^C_2_]GP and [^13^C^15^N]GP reagents used to determine the composition of the fragment ions are shown in the lower right-hand box. An asterisk indicates a heavy isotope label.

7-OC is a α,β-unsaturated ketone (5-en-7-one), and unlike oxysterols/bile acids without an oxo group, will react with GP reagent in the absence of ChOx; hence, this compound and its metabolites possessing a 7-oxo group will be found in the (B) subfraction derivatized with the [^2^H_0_]GP reagent (**Fig. 1B**). Like most GP derivatives, 7-oxo compounds give an intense [M-Py]^+^ ion upon MS^2^ (MS/MS; Fig. 1B); however, MS^3^ ([M]^+^→[M-Py]^+^→) fragmentation patterns for the 7-oxo derivatives are unlike those from compounds with GP derivatization at position C-3 (cf. **Fig. 2B**, C). In contrast to 3-oxo compounds, 7-oxo compounds showed a prominent pattern of fragment ions corresponding to [M-Py-43]^+^, [M-Py-59]^+^, [M-Py-90]^+^, and [M-Py-98]^+^ (*m/z* 412.4, 396.3, 365.3, and 357.3). The suggested structures of these ions are shown in **Fig. 3** and supplemental Fig. S1A. As can be seen in Fig. 3, the neutral losses are associated with the unsaturated diazacyclohexanone ring and water; this is evident by the invariant nature of the neutral loss upon variation of the sterol side chain or by the incorporation of deuterium atoms in the side chain, e.g., as in [25,26,26,26,27,27,27-^2^H_7_]7-OC (cf. Fig. 2B and supplemental Fig. S2A). Definitive identification of the neutral losses was achieved by utilizing [^13^C^15^N] and [^13^C_2_] isotope-labeled GP reagents (Fig. 3). Neutral losses, [M-Py-18]^+^, [M-Py-28]^+^, and [M-Py-61]^+^ common to 7-oxo-5-ene and 3-oxo-4-ene derivatives are illustrated in Fig. 3 and supplemental Fig. S3A.

7-OC is well resolved from its isomer 7α-hydroxycholest-4-en-3-one (7α-HCO) in the 17 min chromatographic gradient and is readily quantified by isotope dilution against [^2^H_7_]7-OC (Fig. 2A), but 26-hydroxy-7-oxocholesterol (26H,7O-C), its CYP27A1 metabolite (28, 29), is only partially resolved from its isomer 7α,26-dihydroxycholest-4-en-3-one (7α,26-diHCO) by the 17 min chromatographic gradient, although it is well resolved from 25-hydroxy-7-oxocholesterol, 7α,12α-dihydroxycholest-4-en-3-one, 7α,24S-dihydroxycholest-4-en-3-one (7α,24S-diHCO), and 7α,25-dihydroxycholest-4-en-3-one (7α,25-diHCO), four further metabolites identified in human plasma. Extending the chromatographic gradient to 37 min provides almost baseline separation of 26H,7O-C from 7α,26-diHCO while maintaining resolution from the other isomers (Fig. 2D). In the absence of an ideal isotope-labeled standard, approximate quantification of 26H,7O-C is performed against the internal standard [^2^H_7_]22R-HCO, taking into account relative response factors.

**Fig. 4.**
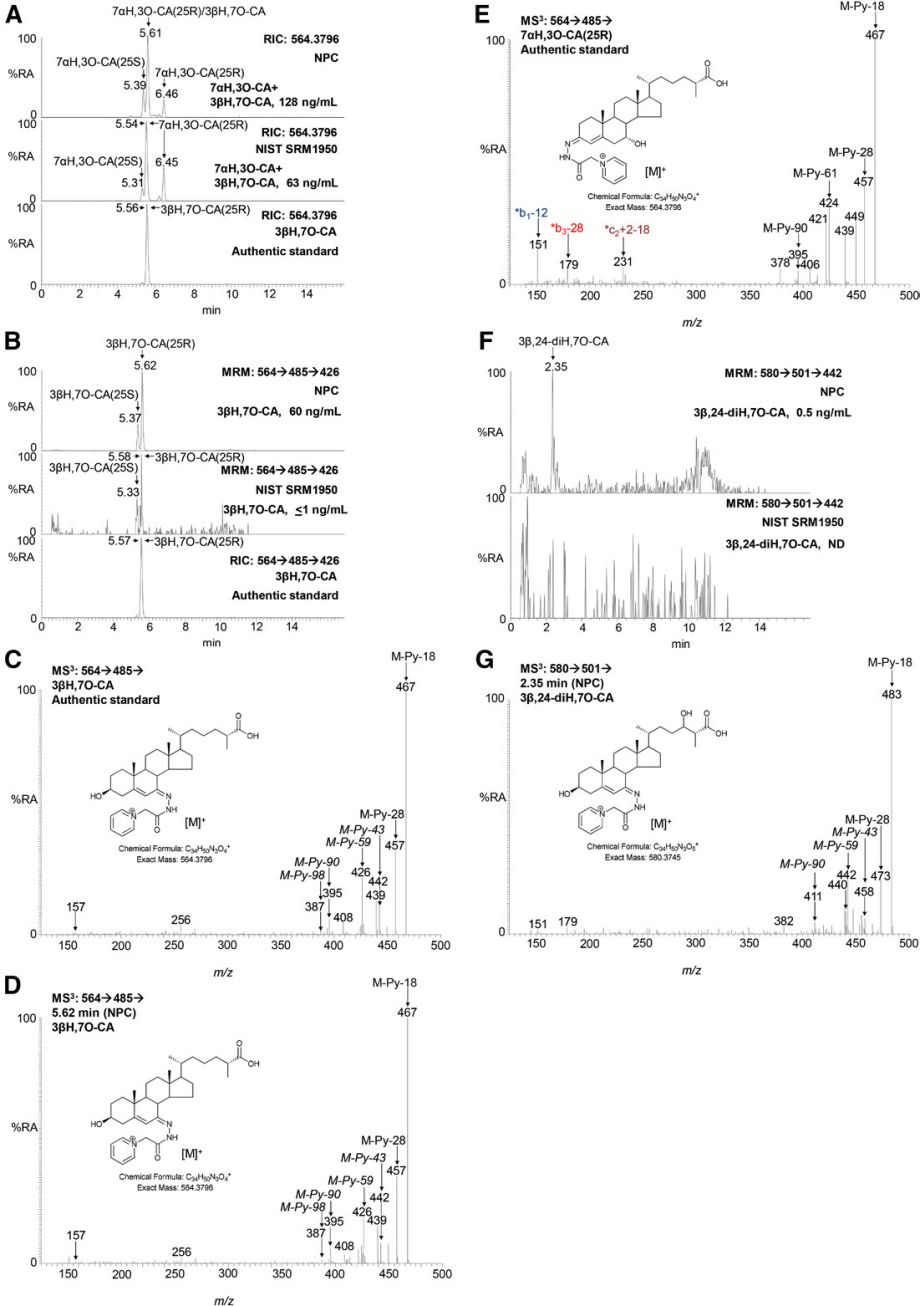
MRM chromatograms [M]^+^→[M-Py]^+^→[M-Py-59]^+^ reveal 3βH,7O-CA and 3β,24-diH,7O-CA in NPC plasma. A: The authentic standard 3βH,7O-CA (bottom) coelutes with 7αH,3O-CA (NPC plasma, top; NIST plasma, middle) in the 17 min chromatographic gradient. B: MRM chromatogram (*m/z* 564.4→485.3→426.3) from an NPC plasma sample (top chromatogram), the NIST control sample (middle chromatogram), and an authentic standard of 3βH,7O-CA (bottom chromatogram). Measured concentrations of 7αH,3O-CA + 3βH,7O-CA and of 3βH,7O-CA alone are given on the right-hand side of the chromatograms A and B, respectively. 25S and 25R epimers of 7αH,3O-CA each give twin peaks corresponding to *syn* and *anti* conformers of the derivative as seen in A. The twin peaks observed in B from NPC and NIST samples probably correspond to 25S and 25R epimers of 3βH,7O-CA. MS^3^ spectra of 3βH,7O-CA authentic standard (C), 3βH,7O-CA from a NPC plasma sample (D), and 7αH,3O-CA(25R) authentic standard (E). F: MRM chromatogram (*m/z* 580.4→501.3→442.3) from an NPC plasma sample (upper) and the NIST plasma sample (lower) generated with the 17 min gradient. Measured concentrations of 3β,24-diH,7O-CA are given in the right-hand corners of the chromatograms. ND, not detected. G: MS^3^ ([M]^+^→[M-Py]^+^→) spectrum underlying the major peak at 2.35 min in F from NPC plasma, identified as of 3β,24-diH,7O-CA. Structures of fragment ions are shown in supplemental Fig. S1B. MS^3^ spectra of 7αH,3O-CA(25R) from NIST plasma, 7α,24S-diH,3O-CA, and 7α,25-diH,3O-CA authentic standards are shown in supplemental Fig. S2H–J. %RA, % relative abundance.

The downstream CYP27A1 metabolite of 26H,7-OC, 3β-hydroxy-7-oxocholest-5-en-26-oic acid (3βH,7O-CA) (29), is not resolved from its isomer 7α-hydroxy-3-oxocholest-4-en-26-oic acid (7αH,3O-CA) in either the 17 or 37 min chromatographic gradients (**Fig. 4A**). However, 3βH,7O-CA gives a MS^3^ fragment ion at *m/z* 426.3 ([M-Py-59]^+^) that is not present in the MS^3^ spectrum of coeluting 7αH,3O-CA (Fig. 4C, E). Thus, by generating a reconstructed-ion chromatogram (RIC) for *m/z* 426.3 ± 0.3 from the MS^3^ spectra, 3βH,7O-CA is revealed (Fig. 4B). Therefore, multiple reaction monitoring (MRM), [M]^+^→[M-Py]^+^→[M-Py-59]^+^, can be used to resolve 3βH,7O-CA from its coeluting isomer 7αH,3O-CA. The fragment-ion at *m/z* 421.3 ([M-Py-64]^+^) is present in the MS^3^ spectrum of 7αH,3O-CA (Fig. 4E), but not 3βH,7O-CA (Fig. 4C); hence, the MRM [M]^+^→[M-Py]^+^→[M-Py-64]^+^ can identify 7αH,3O-CA. By necessity, semiquantification of 3βH,7O-CA was performed utilizing the MRM transition 564.4→485.3→426.3 and reference to an external standard.

In bile acid biosynthesis, side-chain shortening occurs in the peroxisome and proceeds through 24-hydroxylation of the C_27_ acid, dehydrogenation to a 24-carbonyl group, and then β-oxidation to a C_24_ acid (30). The appropriate metabolites from 7-OC are 3β,24-dihydroxy-7-oxocholest-5-en-26-oic acid (3β,24-diH,7O-CA),3β-hydroxy-7,24*-bis*oxocholest-5-en-26-oic acid (3βH,7,24-diO-CA), and 3βH,7O-Δ^5^-BA. Although authentic standards are not available for these metabolites, by analogy to the MS^3^ spectra of 7-OC, 26H,7-OC, and 3βH,7O-CA where standards are available, prominent patterns of distinguishing fragment ions corresponding to the neutral losses [M-Py-43]^+^, [M-Py-59]^+^, [M-Py-90]^+^, and [M-Py-98]^+^ are predicted to be present in the MS^3^ spectra of 3β,24-diH,7O-CA, 3βH,7,24-diO-CA, and 3βH,7O-Δ^5^-BA. Similar to the analysis of 3βH,7O-CA, a MS^3^ RIC for [M-Py-59]^+^ (i.e., *m/z* 442.3 ± 0.3) should reveal 3β,24-diH,7O-CA (Fig. 4F). A fragment ion at *m/z* 442.3 is absent from the MS^3^ spectra of 7α,24S-dihydroxy-3-oxocholest-4-en-(25R)26-oic (7α,24S-diH,3O-CA) and 7α,25-dihydroxy-3-oxocholest-4-en-26-oic (7α,25-diH,3O-CA) acids, two commercially available isomers of 3β,24-diH,7O-CA (supplemental Fig. S2I, J). While for most plasma samples we have analyzed in recent times, the MS^3^ RIC channel for *m/z* 442.3 ± 0.3 is empty (27), when patient samples containing high levels of 7-OC from diseases such as NPC are analyzed, a peak is evident in the MRM chromatogram *m/z* 580.4→501.3→442.3, i.e., [M]^+^→[M-Py]^+^→[M-Py-59]^+^ (Fig. 4F), and the underlying MS^3^ spectrum is compatible with that predicted for 3β,24-diH,7O-CA (Fig. 4G). In the extended chromatographic gradient of 37 min, presumptively identified 3β,24-diH,7O-CA is resolved from isomeric dihydroxy-3-oxocholest-4-en-26-oic acids (supplemental Fig. S2K). Semiquantitative measurements of 3β,24-diH,7O-CA are made using the extended chromatographic gradient assuming the same response factor as for 7α,24-diH,3O-CA and using the internal standard [^2^H_7_]22R-HCO.

**Fig. 5.**
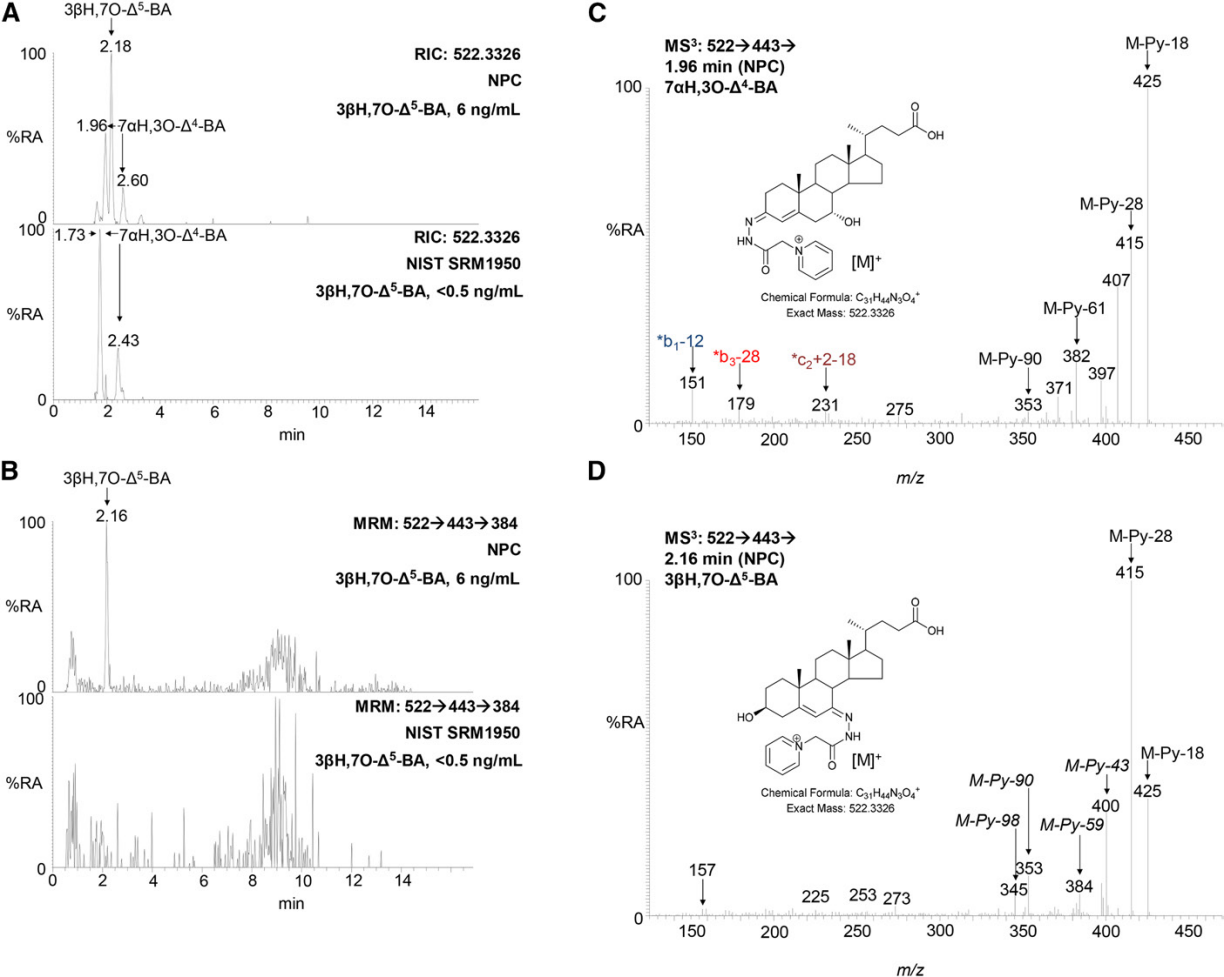
The MRM chromatogram [M]^+^→[M-Py]^+^→[M-Py-59]^+^ reveals 3βH,7O-Δ^5^-BA in samples rich in 7-OC. A: RICs for *m/z* 522.3326 ± 5 ppm corresponding to 3βH,7O-Δ^5^-BA and its isomer 7αH,3O-Δ^4^-BA in NPC (upper) and NIST (lower) plasma samples. B: MRM *m/z* 522.3→443.3→384.3 chromatograms from an NPC (upper) and NIST (lower) plasma sample. Measured concentrations of 3βH,7O-Δ^5^-BA are given in the right-hand corners of the chromatograms. The chromatograms were recorded on different days resulting in an offset in retention time of 0.2 min in the early eluting peaks. MS^3^ ([M]^+^→[M-Py]^+^→) spectra of the compounds underlying the chromatographic peaks eluting at (C) 1.96 min (7αH,3O-Δ^4^-BA) in chromatogram A and (D) 2.16 min (3βH,7O-Δ^5^-BA) in chromatogram B. %RA, % relative abundance.

To date in none of the plasma samples we have analyzed have we observed any chromatographic peaks compatible with 3βH,7,24-diO-CA. In contrast, as with 3β,24-diH,7O-CA, an MS^3^ RIC for [M-Py-59]^+^ (*m/z* 384.3) reveals 3βH,7O-Δ^5^-BA in plasma samples from patients with elevated 7-OC (**Fig. 5B**). 3βH,7O-Δ^5^-BA is clearly resolved from its isomer 7α-hydroxy-3-oxochol-4-enoic acid (7αH,3O-Δ^4^-BA) in the 17 min chromatographic gradient (Fig. 5A). Semiquantitative measurements are made for 3βH,7O-Δ^5^-BA using the extended chromatographic gradient assuming the same response factor as for 7αH,3O-Δ^4^-BA and using the internal standard [^2^H_7_]22R-HCO.

While GP-derivatized sterols with a 7-hydroxy-3-oxo-4-ene structure give a characteristic pattern of ring fragment ions at *m/z* 151.1 (*b_1_-12), 177.1 (*b_2_), 179.1 (*b_3_-28), and 231.1, (*c_2_+2-18) (Figs. 2C, E, 4E, 5C) (31), sterols with a 3β-hydroxy-7-oxo-5-ene structure give a minor fragment-ion at *m/z* 157.1 which probably consists of the unsaturated diazacyclohexanone ring and remnants of the B-ring (Figs. 2B, 4C, D, 5D). This ion is only minor and of limited diagnostic value.

### Semiquantitative measurements

Using the methodology described, other than for 7-OC, where an isotope-labeled standard is available, i.e., [^2^H_7_]7-OC, we can only make approximate or semiquantitative measurements. However, as all the 7-oxo compounds, except 3βH,7O-CA, are resolved from their 3-oxo isomers in either the 17 or 37 min chromatographic gradients, quantification is possible using the isotope-labeled internal standard [^2^H_7_]22R-HCO. In the absence of authentic standards, 3β,24-diH,7O-CA and 3βH,7O-Δ^5^-BA were quantified assuming the same response factors as for their structural analogs 7α,24-diH,3O-CA and 7αH,3O-Δ^4^-BA. As 3βH,7O-CA was not chromatographically resolved from 7αH,3O-CA, the MRM [M^+^]→[M-Py]^+^→[M-Py-59]^+^ was used for quantification of the former isomer.

### 3β,5α,6β-Trihydroxysterols

**Fig. 6.**
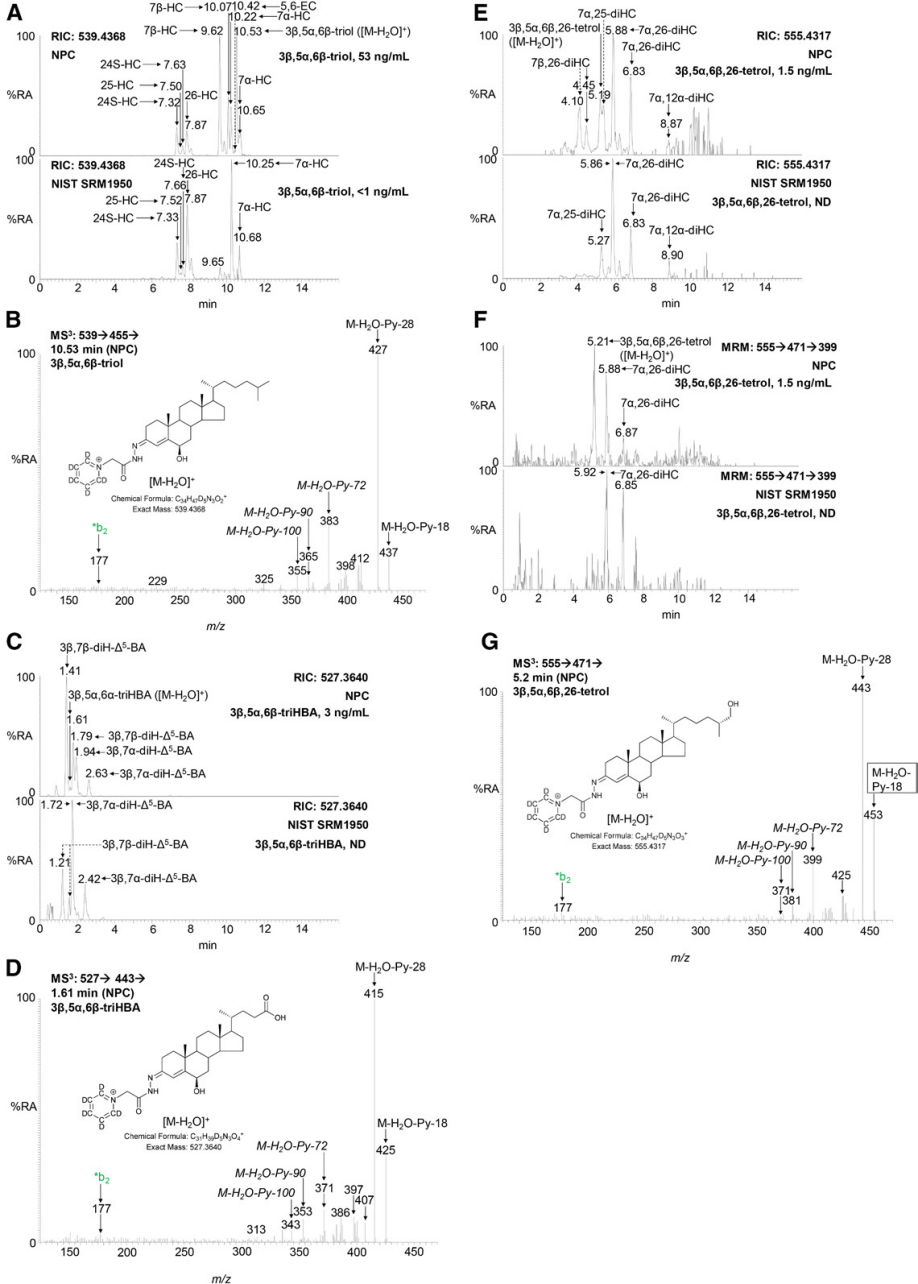
3β,5α,6β-triol, 3β,5α,6β-triHBA, and 3β,5α,6β,26-tetrol give [M-H_2_O-Py-72]^+^ and [M-H_2_O-Py-100]^+^ neutral-loss fragment ions in their MS^3^ ([M-H_2_O]^+^→[M-H_2_O-Py]^+^→) spectra. A: RIC, *m/z* 539.4368 ± 5 ppm, demonstrating chromatographic separation of 3β,5α,6β-triol ([M-H_2_O]^+^ ions) from hydroxycholesterols ([M]^+^ ions) and 5,6-EC ([M]^+^ ions) in NPC (upper) and NIST (lower) plasma samples. Measured concentrations of 3β,5α,6β-triol are given in the right-hand corners of the chromatograms. Monohydroxycholesterols give *syn* and *anti* conformers of the GP derivative, resulting in twin peaks. B: MS^3^ ([M-H_2_O]^+^→[M-H_2_O-Py]^+^→) spectrum of 3β,5α,6β-triol from an NPC plasma sample. C: RIC of *m/z* 527.3640 ± 5 ppm demonstrating chromatographic separation of 3β,5α,6β-triHBA [M-H_2_O]^+^ from 3β,7β-diH-Δ^5^-BA ([M]^+^ ions) and 3β,7α-diH-Δ^5^-BA ([M]^+^ ions) in NPC (upper) and NIST (lower) plasma samples. Measured concentrations of 3β,5α,6β-triHBA are given in the right-hand corners of the chromatograms. Both diH-Δ^5^-BA isomers give twin chromatographic peaks. The chromatograms were recorded on different days, resulting in a retention time shift of ∼0.2 min. D: MS^3^ ([M-H_2_O]^+^→[M-H_2_O-Py]^+^→) spectrum of 3β,5α,6β-triHBA in an NPC plasma sample. E: RIC for *m/z* 555.4317 corresponding to the [M-H_2_O]^+^ ion of cholestanetetrols and the [M]^+^ ion of dihydroxycholesterols from NPC (upper) and NIST (lower) plasma samples. Measured concentrations of 3β,5α,6β,26-tetrol are given in the right-hand corners of the chromatograms. F: RIC for the MRM transition *m/z* 555.4→471.4→399.3 corresponding to [M-H_2_O]^+^→[M-H_2_O-Py]^+^→[M-H_2_O-Py-72]^+^ for cholestanetetrols from the NPC (upper) and NIST (lower) plasma samples. G: MS^3^ spectrum of the peak eluting at 5.2 min in the NPC plasma sample. See supplemental Fig. S4A and B for assignment of fragment-ions. MS^3^ spectra of authentic standards of [^2^H_7_]3β,5α,6β-triol, 3β,5α,6β-triol, and 5α,6-EC are shown in supplemental Fig. S5C–E. %RA, % relative abundance.

**Fig. 7.**
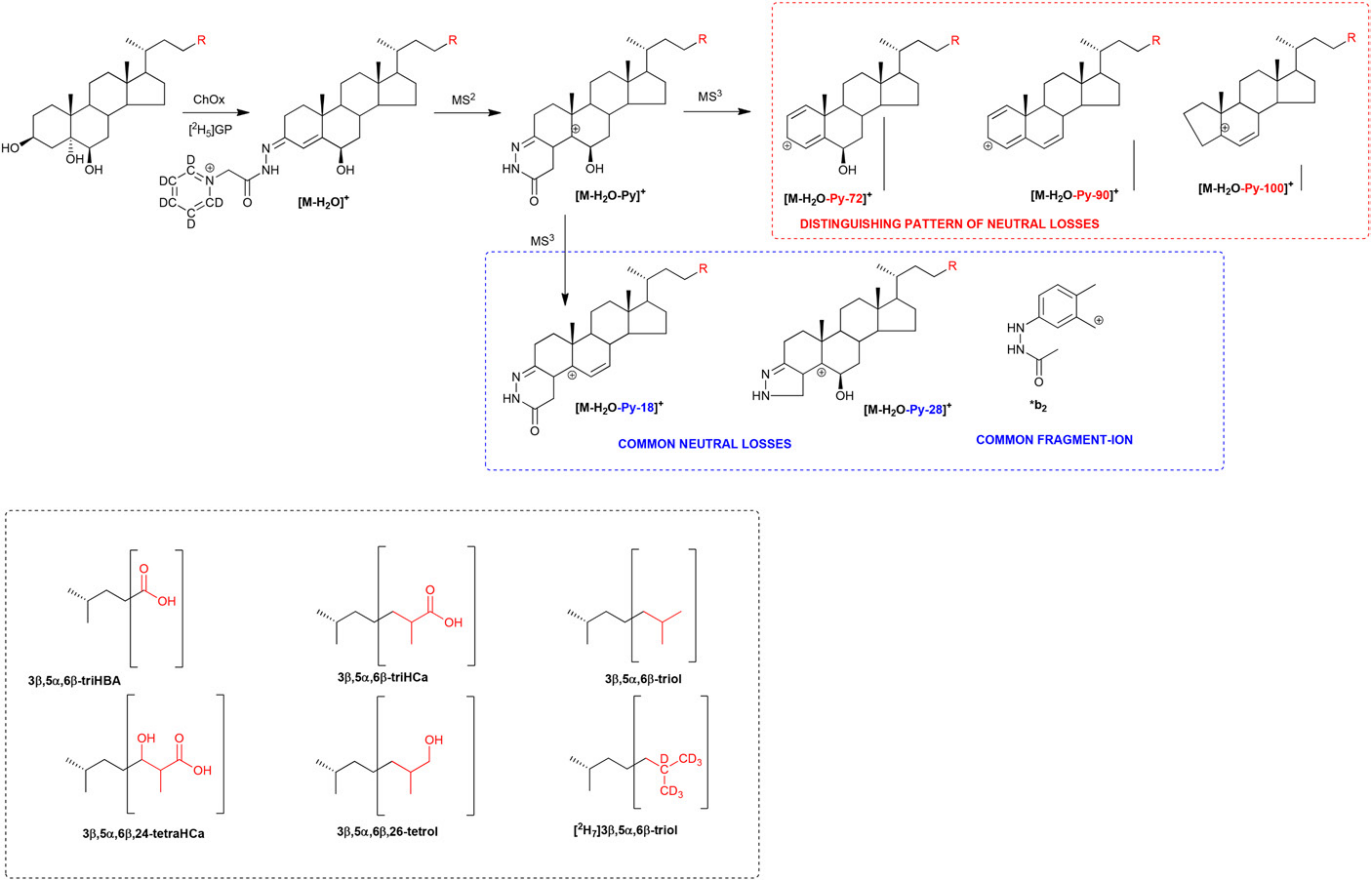
Patterns of MS^3^ neutral losses which distinguish between, or are common to, 3β,5α,6β-triol-containing and 3β,7-dihydroxy-5-ene sterols. The pattern of neutral losses shown in the red box distinguishes between [M-H_2_O]^+^ ions of 3β,5α,6β-triols from [M]^+^ ions of 3β,7-dihydroxy-5-ene sterols of identical mass. Neutral losses/fragment ion shown in the blue box are common to both structures. Structures of R groups are shown within brackets in the lower left-hand box.

Sterols with a 3β-hydroxy group and a planar A/B ring system are substrates for ChOx; these include cholest-5-en-3β-ols and 5α-cholestan-3β-ols (32). 3β,5α,6β-triol is planar and becomes oxidized at C-3 and also dehydrated through elimination of the 5α-hydroxy group. The dehydration reaction does not go to completion under our experimental conditions, so, after derivatization, the GP derivatized triol is observed as both [M]^+^ and [M-H_2_O]^+^ ions (Fig. 1A) (33), with the [M-H_2_O]^+^ ion giving the stronger signal and more informative MS^3^ spectrum (**Fig. 6B**). In fact, the MS^3^ ([M-H_2_O]^+^→[M-H_2_O-Py]^+^→) spectrum of the 3β,5α,6β-triol is identical to the MS^3^ ([M]^+^→[M-Py]^+^→) spectrum of cholest-4-ene-3β,6β-diol (6β-HC), confirming dehydration through loss of the 5α-hydroxy group. An unusually prominent fragment-ion observed in the MS^3^ ([M-H_2_O]^+^→[M-H_2_O-Py]^+^→) spectrum of 3β,5α,6β-triol is at *m/z* 383.3, corresponding to [M-H_2_O-Py-72]^+^ (Fig. 6B; see also **Fig. 7**). A second unusual neutral-loss [M-H_2_O-Py-100]^+^ gives a fragment-ion at *m/z* 355.3. Both fragment ions are elevated by 7 Da in the spectrum of the [25,26,26,26,27,27,27-^2^H_7_] analog, as is the [M-H_2_O-Py-90]^+^ fragment ion (see supplemental Figs. S4A, S5C, D). The availability of [^2^H_7_]3β,5α,6β-triol allows quantification by isotope dilution utilizing RICs for [M-H_2_O]^+^ ions. Similar to 3β,5α,6β-triol, 3β,5α,6β-triHBA, the end product of 3β,5α,6β-triol metabolism (15, 16), gives [M]^+^ and [M-H_2_O]^+^ ions after GP derivatization, the latter of which is dominant. The MS^3^ ([M-H_2_O]^+^→[M-H_2_O-Py]^+^→) spectrum of 3β,5α,6β-triHBA shows a prominent [M-H_2_O-Py-72]^+^ fragment-ion at *m/z* 371.3, [M-H_2_O-Py-90]^+^ at *m/z* 353.2, and the unusual neutral-loss [M-H_2_O-Py-100]^+^ at *m/z* 343.3 (Fig. 6D; see also supplemental Figs. S4B and S5I). The particularly prominent neutral loss fragment ion [M-H_2_O-Py-72]^+^ is common to MS^3^ spectra of both 3β,5α,6β-triol and 3β,5α,6β-triHBA and can potentially be used to identify further metabolites with a 3β,5α,6β-trihydroxy structure via [M-H_2_O]^+^→[M-H_2_O-Py]^+^→[M-H_2_O-Py-72]^+^ MRM chromatograms (see below). In the absence of an isotope-labeled standard, approximate quantification of 3β,5α,6β-triHBA is made against the internal standard [^2^H_7_]24R/S-HC, taking into account relative response factors.

In plasma samples high in 3β,5α,6β-triol (e.g., NPC), a new peak is evident in the RIC for the [M-H_2_O]^+^ ion of cholestanetetrols (*m/z* 555.4317 ± 5 ppm, 5.19 min in Fig. 6E), which is not seen in control plasma. With both the 17 and 37 min chromatographic gradients, this peak is only partially resolved from the [M]^+^ ion of 7α,25-dihydroxycholesterol (7α,25-diHC) which has an identical mass. However, chromatographic resolution is sufficiently good to generate an MS^3^ (*m/z* 555.4→471.4→) spectrum from the apex of the new peak, which is entirely compatible with that expected for the [M-H_2_O]^+^ ion of cholestane-3β,5α,6β,26-tetrol (3β,5α,6β,26-tetrol), showing a prominent fragment-ion at *m/z* 399.3, i.e., [M-H_2_O-Py-72]^+^; a distinct ion at *m/z* 381.3, i.e., [M-H_2_O-Py-90]^+^; and a minor fragment at *m/z* 371.3, i.e., [M-H_2_O-Py-100]^+^ (Fig. 6G; see also Fig. 7). The MS^3^ (*m/z* 555.4→471.4→) spectrum of closely eluting 7α,25-diHC does not give a fragment ion at *m/z* 399.3 or 371.3 (supplemental Fig. S5L), so by generating a RIC for the fragment ion *m/z* 399.3 from the MS^3^ (*m/z* 555.4→471.4→) chromatogram, 3β,5α,6β,26-tetrol ([M-H_2_O]^+^) is resolved from 7α,25-diHC ([M]^+^, Fig. 6F). Other isomers of 7α,25-diHC ([M]^+^), i.e., the dihydroxycholesterols (diHC) 7α,12α-diHC, 7α,24S-diHC, 7α,26-diHC, 7β,25-diHC, and 7β,26-diHC, are all chromatographically resolved from 3β,5α,6β,26-tetrol ([M-H_2_O]^+^). It is only possible to make semiquantitative measurement of 3β,5α,6β,26-tetrol in the absence of an authentic standard and its incomplete chromatographic resolution from 7α,25-diHC. Semiquantification is made based on the [M-H_2_O]^+^ of 3β,5α,6β,26-tetrol against the internal standard [^2^H_6_]7α,25-diHC.

CYP27A1 is the enzyme likely to introduce the (25R)26-hydroxy group to the sterol side chain. This enzyme could then oxidize the primary alcohol to a carboxylic acid to give 3β,5α,6β-trihydroxycholestan-(25R)26-oic acid (3β,5α,6β-triHCa). As discussed above, peroxisomal side-chain shortening of C_27_ acids proceeds through C-24 hydroxylation, C-24 dehydrogenation, and β-oxidation to generate 3β,5α,6β-triHBA as the ultimate product. The relevant pathway intermediates would be 3β,5α,6β,24-tetrahydroxycholestan-26-oic acid (3β,5α,6β,24-tetraHCa) and 3β,5α,6β-trihydroxy-24-oxocholestan-26-oic acid (3β,5α,6β-triH,24O-Ca). By analogy to 3β,5α,6β-triol, 3β,5α,6β,26-tetrol, and 3β,5α,6β-triHBA, 3β,5α,6β-triHCa, 3β,5α,6β,24-tetraHCa, and 3β,5α,6β-triH,24O-Ca should give [M]^+^ and [M-H_2_O]^+^ products upon ChOx treatment and GP derivatization, and the MS^3^ spectra of the [M-H_2_O]^+^ ions ([M-H_2_O]^+^→[M-H_2_O-Py]^+^→]) are predicted to show neutral-loss fragment-ions [M-H_2_O-Py-72]^+^, [M-H_2_O-Py-90]^+^, and [M-H_2_O-Py-100]^+^.

**Fig. 8.**
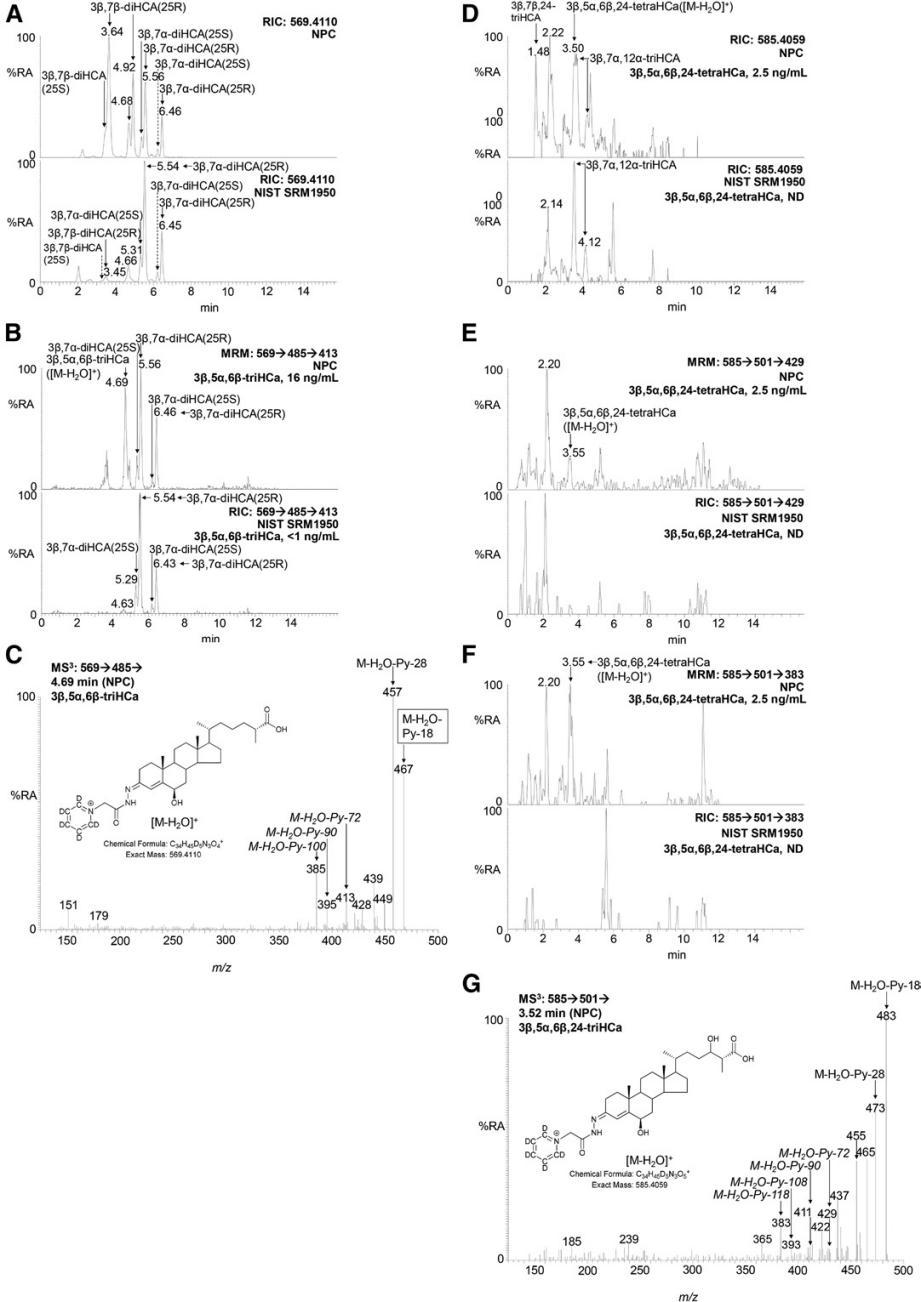
Identification of 3β,5α,6β-triHCa and 3β,5α,6β,24-tetraHCa in plasma samples rich in 3β,5α,6β-triol. A: RIC of *m/z* 569.4110 ± 5 ppm corresponding to [M-H_2_O]^+^ and [M]^+^ ions of 3β,5α,6β-triHCa and dihydroxycholestenoic acids, respectively, from plasma from a patient with NPC (upper) and the NIST control plasma (lower). 3β,7α-dihydroxycholest-5-en-(25R)26-oic acid and 3β,7β-diHCA appear as 25S and 25R epimers, and both give twin peaks due to *syn* and *anti* conformers of the GP derivative. The NPC plasma was analyzed on a different day from the NIST plasma samples, resulting in a 0.1–0.2 min offset in the earlier eluting chromatographic peaks. B: RIC for the MRM transitions 569.4→485.3→413.3 in plasma from a patient with NPC (upper) and the NIST control plasma (lower). Measured concentrations of 3β,5α,6β-triHCa are given on the right-hand side of the chromatograms. C: MS^3^ ([M-H_2_O]^+^→[M-Py-H_2_O]^+^→) spectrum from the compound underlying the chromatographic peak at 4.69 min in the NPC chromatogram in B. See supplemental Fig. S4B for a description of fragment ions. D: RIC of *m/z* 585.4059 ± 5 ppm corresponding to [M-H_2_O]^+^ of 3β,5α,6β,24-tetraHCa and [M]^+^ of trihydroxycholestenoic acids in NPC (upper) and NIST control plasma (lower). MRM chromatograms *m/z* 585.4→501.3→429.3 (E) and *m/z* 585.4→501.3→383.3 (F), revealing 3β,5α,6β,24-tetraHCa in NPC (upper), but not the NIST control plasma (lower). Measured concentrations of 3β,5α,6β,24-tetraHCa are given on the right-hand side of the chromatograms. The NPC plasma was analyzed on a different day from the NIST plasma samples resulting in a 0.1–0.2 min offset in the earlier eluting chromatographic peaks. G: MS^3^ ([M-H_2_O]^+^→[M-H_2_O-Py]^+^→) spectrum of 3β,5α,6β,24-tetraHCa in NPC plasma. % RA, % relative abundance.

For 3β,5α,6β-triHCa, the [M-H_2_O-Py-72]^+^ ion has an *m/z* of 413.3 (see supplemental Fig. S4B). A RIC for the MRM transition [M-H_2_O]^+^→[M-Py-H_2_O]^+^→[M-H_2_O-Py-72]^+^ (*m/z* 569.4→485.3→413.3) reveals a new chromatographic peak in samples where the concentration of the 3β,5α,6β-triol is high (e.g., NPC), which is at, or below, the detection limit in normal plasma samples (**Fig. 8B**). The MS^3^ ([M-H_2_O]^+^→[M-H_2_O-Py]^+^→) spectrum underlying the new chromatographic peak in plasma samples rich in 3β,5α,6β-triol is compatible with that predicted for 3β,5α,6β-triHCa, showing the predicted neutral-loss fragment ions [M-H_2_O-Py-72]^+^, [M-H_2_O-Py-90]^+^, and [M-H_2_O-Py-100]^+^ and is thus assigned to this acid (Fig. 8C; see also supplemental Figs. S4B, S5N). This new chromatographic peak does, however, coelute with that of the [M]^+^ ion of 3β,7β-dihydroxycholest-5-en-(25S)26-oic acid [3β,7β-diHCA(25S)] and a second oxysterol with a probable 3β,22,25-trihydroxycholest-5-en-24-one structure, but neither compound gives a fragment ion at *m/z* 413.3 in their MS^3^ ([M]^+^→[M-Py]^+^→) spectra (supplemental Fig. 5O), unlike well-resolved 3β,7α-dihydroxycholest-5-en-(25S)26-oic and 3β,7α-dihydroxycholest-5-en-(25R)26-oic acids, where this ion is more abundant (supplemental Fig. S5P). In fact, the 25R and 25S epimers give identical MS^3^ spectra, but are chromatographically resolved. In the absence of an authentic standard of 3β,5α,6β-triHCa, only semiquantification is possible. This can be made using the RIC for the [M-H_2_O]^+^ ion in samples where coeluting compounds 3β,7β-diHCA(25S) and 3β,22,25-trihydroxycholest-5-en-24-one are minor (and assuming a similar response factor to 3β,5α,6β-triHBA), then determining an appropriate response factor for the MRM chromatogram 569.4→485.3→413.3 and using this for quanti­fication of other samples.

For 3β,5α,6β,24-tetraHCa, the [M-H_2_O-Py-72]^+^ fragment ion has an *m/z* of 429.3 (supplemental Fig. S4B). In the RIC for the MRM transition [M-H_2_O]^+^→[M-Py-H_2_O]^+^→[M-H_2_O-Py-72]^+^ (*m/z* 585.4→501.3→429.3) from samples where 3β,5α,6β-triol is abundant, new peaks appears in both the 17 min (Fig. 8E) and 37 min chromatograms. The underlying MS^3^ spectrum of the new peak at 3.5 min in an NPC plasma sample (Fig. 8G; see also supplemental Fig. S5Q) shows a minor fragment ion at *m/z* 429.3 and a more prominent ion at *m/z* 383.3, the former corresponding to [M-H_2_O-Py-72]^+^ and the latter [M-H_2_O-Py-118], a dehydration product of [M-H_2_O-Py-100]^+^ (see supplemental Fig. S4B). Both fragment ions are essentially absent from MS^3^ spectra of the closely eluting peak (Fig. 8D), annotated as 3β,7α,12α-trihydroxycholest-5-en-(25R)26-oic acid (supplemental Fig. S5R) and its isomers 3β,7α,24-trihydroxycholest-5-en-(25R)26-oic and 3β,7α,25-trihydroxycholest-5-en-26-oic acid. The MRM chromatogram [M-H_2_O]^+^→[M-Py-H_2_O]^+^→[M-H_2_O-Py-118]^+^ provides even clearer definition of 3β,5α,6β,24-tetraHCa (Fig. 8F). The chromatographic peak for the [M-H_2_O]^+^ ion of 3β,5α,6β,24-tetraHCa is sufficiently resolved in the 37 min gradient to allow semiquantification against the internal standard [^2^H_7_]24R/S-HC and by assuming a response factor similar to 3β,5α,6β-triHBA.

### Semiquantitative measurements

Other than for 3β,5α,6β-triol itself, for which the [^2^H_7_] analog is available, we can only make approximate or semiquantitative measurements of 3β,5α,6β-triol containing sterols. An authentic standard of 3β,5α,6β-triHBA is available, and thus can be used to give approximate quantification. Other than 3β,5α,6β-triHCa, all of the 3β,5α,6β-triol-con­taining sterols are sufficiently chromatographically resolved from similarly derivatized sterols to allow semiquantification against added internal standards.

## DISCUSSION

When 7-OC or 3β,5α,6β-triol is abundant in a sample, whether formed enzymatically or through radical reactions, the analyst should consider the possibility of the presence of downstream metabolites. An absence may indicate that the primary metabolites are formed ex vivo, while a presence will indicate formation in vivo or perhaps from the diet or environment. By taking plasma samples from patients with the lysosomal storage disorder NPC as an example, we illustrate how metabolites of 7-OC and 3β,5α,6β-triol leading to bile acids 3βH,7O-Δ^5^-BA and 3β,5α,6β-triHBA, respectively, can be identified.

Considering metabolites with a 7-oxo-5-ene structure, the fragment ion resulting from an [M-Py-59]^+^ neutral loss (Fig. 3) and appearing in MS^3^ ([M]^+^→[M-Py]^+^→) spectra is characteristic and valuable for metabolite identification via appropriate MRM ([M]^+^→[M-Py]^+^→[M-Py-59]^+^) RICs (Figs. 4B, F, 5B). The [M-Py-59]^+^ fragment ion is likely a radical cation stabilized by delocalization across a conjugated system from C3–C7 and two nitrogen atoms (Fig. 3). With respect to sterols containing a 3β,5α,6β-triol structure, treatment with ChOx and GP derivatization leads to dehydration through loss of the 5α-hydroxy group and formation of an [M-H_2_O]^+^ ion. MS^3^ ([M-H_2_O]^+^→[M-H_2_O-Py]^+^→) leads to a characteristic neutral-loss fragment ion [M-H_2_O-Py-72]^+^ (Fig. 7). Again, the appropriate MRM, [M-H_2_O]^+^→[M-H_2_O-Py]^+^→[M-H_2_O-Py-72]^+^, can lead to the identification of 3β,5α,6β-triol-containing metabolites (Figs. 6F, 8B, E). The [M-H_2_O-Py-72]^+^ fragment ion is likely stabilized through delocalization of positive charge across the conjugated double bonds in the A-ring (Fig. 7). Besides giving the [M-H_2_O]^+^ ion, both 3β,5α,6β-triol and 3β,5α,6β-triHBA give an [M]^+^ ion. However, the absence of A/B-ring unsaturation leads to MS^3^ ([M]^+^→[M-Py]^+^→) spectra, which are less structurally characteristic, making identification of intermediate metabolites difficult (supplemental Fig. S5A, H).

Although the primary aim of this work was identification of 7-oxo-5-ene- and 3β,5α,6β-triol-containing metabolites, approximate or semiquantitative measurements can also be made. Accurate quantification, however, awaits further synthesis of authentic standards and their isotope-labeled analogs.

## Supplementary Material

Supplemental Data
