# Supplementary material for: Identification of unusual oxysterols and bile acids with 7-oxo or 3β,5α,6β-trihydroxy functions in human plasma by charge-tagging mass spectrometry with multistage fragmentation
Source: J Lipid Res. 2018 Apr 6;59(6):1058–70. doi: 10.1194/jlr.D083246 (PMC5983402; doi:10.1194/jlr.D083246)
Supplement: Supplemental Data [file supp_59_6_1058__index.html]

Identification Of Unusual Oxysterols And Bile acids With 7-Oxo Or 3β,5α,6β-Trihydroxy Functions In Human Plasma By Charge-Tagging Mass Spectrometry With Multistage Fragmentation — Identification of unusual oxysterols and bile acids with 7-oxo or 3β,5α,6β-trihydroxy functions in human plasma by charge-tagging mass spectrometry with multistage fragmentation — Supplemental Data 

# Identification of unusual oxysterols and bile acids with 7-oxo or 3β,5α,6β-trihydroxy functions in human plasma by charge-tagging mass spectrometry with multistage fragmentation

## Supplemental Data

- Supplemental Figures (.pdf, 1.5 MB) - Supplemental Figures
- Supplemental Table (.xlsx, 18 KB) - Supplemental Table
