# Supplementary material for: Identification of unusual oxysterols and bile acids with 7-oxo or 3β,5α,6β-trihydroxy functions in human plasma by charge-tagging mass spectrometry with multistage fragmentation
Source: J Lipid Res. 2018 Apr 6;59(6):1058–70. doi: 10.1194/jlr.D083246 (PMC5983402; doi:10.1194/jlr.D083246)
Supplement: Supplemental Data [file 10.1194_D083246_jlr.D083246-1.pdf]

Supplemental Data

Identification of unusual oxysterols and bile acids with 7-oxo or 3β,5α,6β-trihydroxy functions in human plasma by charge-tagging mass spectrometry with multistage fragmentation

William J. Griffiths[1]*, Ian Gilmore[1], Eylan Yutuc[1], Jonas Abdel-Khalik[1], Peter J. Crick[1], Thomas Hearn[1], Alison Dickson[1], Brian W. Bigger[2], Teresa Hoi-Yee Wu[3], Anu Goenka[3], Arunabha Ghosh[3], Simon A. Jones[3] and Yuqin Wang[1]*

[1]Swansea University Medical School, ILS1 Building, Singleton Park, Swansea SA2 8PP, Wales, UK

[2]Stem Cell & Neurotherapies, Division of Cell Matrix Biology and Regenerative Medicine, Stopford Building, Oxford Road, University of Manchester, Manchester M13 9PT, UK

[3]Manchester Centre for Genomic Medicine, 6th floor, St Mary's Hospital, Central Manchester Foundation Trust, University of Manchester, Oxford Road, Manchester M13 9WL, UK

Figure Captions

Supplemental Figure S1. MS^3 neutral-losses characteristic of [$^2H_0$]GP derivatised 7-oxo-5-ene sterols. (A) 7-OC; [$^2H_7$]7-OC; 26H,7-OC. (B) 3βH,7O-CA; 3β,24-diH,7O-CA; 3βH,7O-Δ^5-BA.

Supplemental Figure S2. MS^3 ($[M]^+ \rightarrow [M-Py]^+ \rightarrow$) spectra of (A) [$^2H_7$]7-OC authentic standard, (B) 7-OC authentic standard, (C) 7α-HCO authentic standard, (D) 26H,7O-C authentic standard, (E) 7α,26-diHCO authentic standard, (F) 7α,25-diHCO authentic standard, (G) 7α,24S-diHCO authentic standard, (H) 7αH,3O-CA(25R) from NIST plasma, (I) 7α,24S-diH,3O-CA authentic standard, (J) 7α,25-diH,3O-CA authentic standard and (K) 37 min chromatogram demonstrating the separation of 3β,24-diH,7O-CA from its isomers.

Supplemental Figure S3. MS^3 neutral-losses common to [$^2H_0$]GP derivatised 7-oxo-5-ene and 3-oxo-4-ene sterols. (A) 7α-HCO; [$^2H_7$]7α-HCO; 7-OC; [$^2H_7$]7-OC. (B) 7α,26-diHCO; 26H,7O-C; 7αH,3O-CA; 3βH,7O-CA. (C) 7α,24-diH,3O-CA, 3β,24-diH,7O-CA; 7αH,3O-Δ^4-BA; 3βH,7O-Δ^5-BA.

Supplemental Figure S4. MS^3 ($[M-H_2O]^+ \rightarrow [M-H_2O-Py]^+ \rightarrow$]) neutral-loss fragment-ions characteristic of [$^2H_5$]GP derivatised sterols with a 3β,5α,6β-triol function. (A) 3β,5α,6β-triol, [$^2H_7$]3β,5α,6β-triol and 3β,5α,6β,26-tetrol. (B) 3β,5α,6β-triHCa, 3β,5α,6β,24-tetraHCa, 3β,5α,6β-triHBA.

Supplemental Figure S5. MS^3 ($[M]^+ \rightarrow [M-Py]^+ \rightarrow$) spectra of (A) [$^2H_7$]3β,5α,6β-triol authentic standard and (B) 3β,5α,6β-triol from NPC plasma. MS^3 ($[M-H_2O]^+ \rightarrow [M-H_2O-Py]^+ \rightarrow$) spectra of (C) [$^2H_7$]3β,5α,6β-triol authentic standard and (D) 3β,5α,6β-triol authentic standard. MS^3 ($[M]^+ \rightarrow [M-Py]^+ \rightarrow$) spectra of (E) 5α,6-EC authentic standard, (F) 5,6-EC from NPC plasma, (G) 7α-HC from NPC plasma and (H) 3β,5α,6β-triHBA authentic standard. MS^3 ($[M-H_2O]^+ \rightarrow [M-H_2O-Py]^+ \rightarrow$) spectrum of (I) 3β,5α,6β-triHBA authentic standard. MS^3 ($[M]^+ \rightarrow [M-Py]^+ \rightarrow$) spectra of (J) 3β,7β-diH-Δ^5-BA, (K) 3β,7α-diH-Δ^5-BA, (L) 7α,25-diHC and (M) 7α,26-diHC from NPC or NIST plasma. (N) MS^3 ($[M-H_2O]^+ \rightarrow [M-H_2O-Py]^+ \rightarrow$) spectrum of 3β,5α,6β-triHCa from plasma from a patient with lysosomal acid lipase deficiency. MS^3 ($[M]^+ \rightarrow [M-Py]^+ \rightarrow$) spectra of (O) 3β,7β-diHCA(25R) authentic standard and (P) 3β,7α-diHCA(25R) from NPC plasma. (Q) MS^3 ($[M-H_2O]^+ \rightarrow [M-H_2O-Py]^+ \rightarrow$) spectrum of 3β,5α,6β,24-triHCa from plasma from a patient with lysosomal acid lipase deficiency. (R) MS^3 ($[M]^+ \rightarrow [M-Py]^+ \rightarrow$) spectrum of 3β,7α,12α-triHCA from NIST plasma.

Supplemental Figure S1

S1A

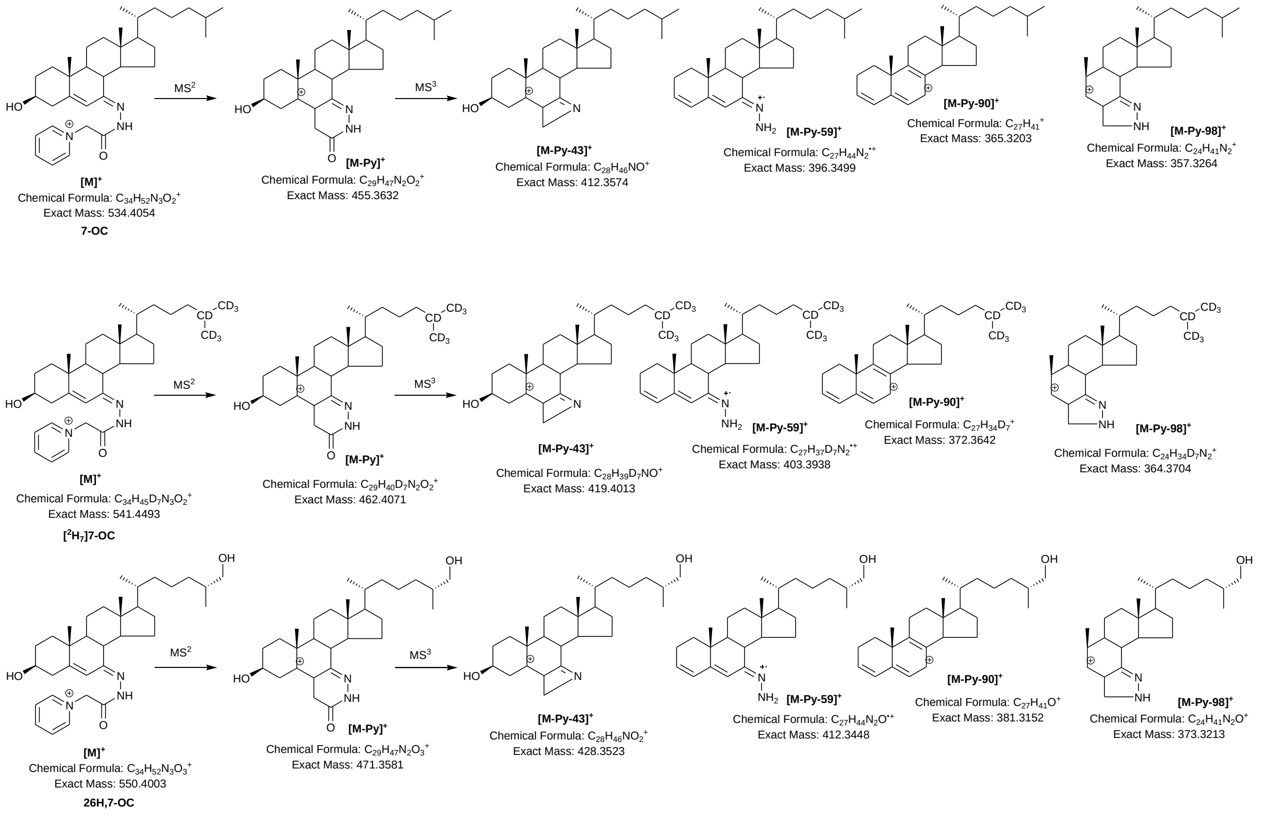

S1B

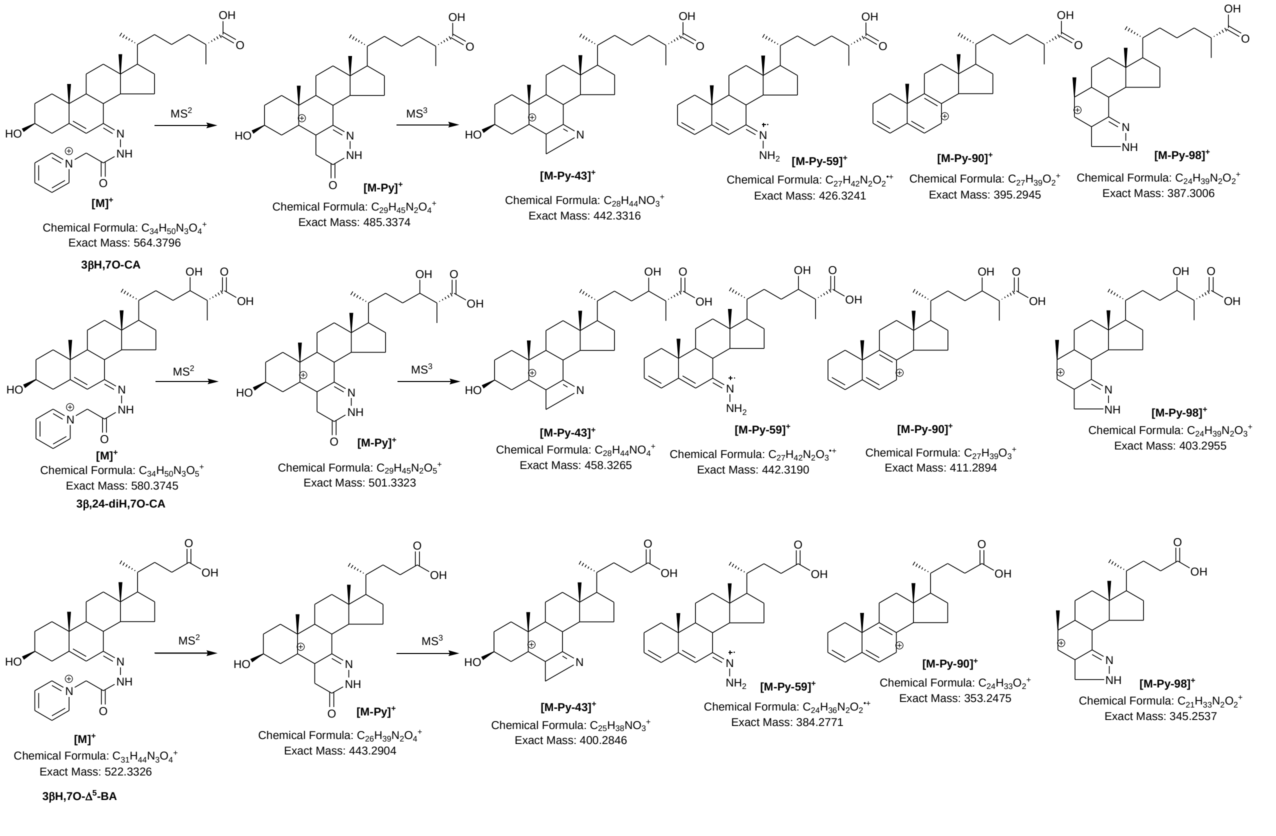

Supplemental Figure S2

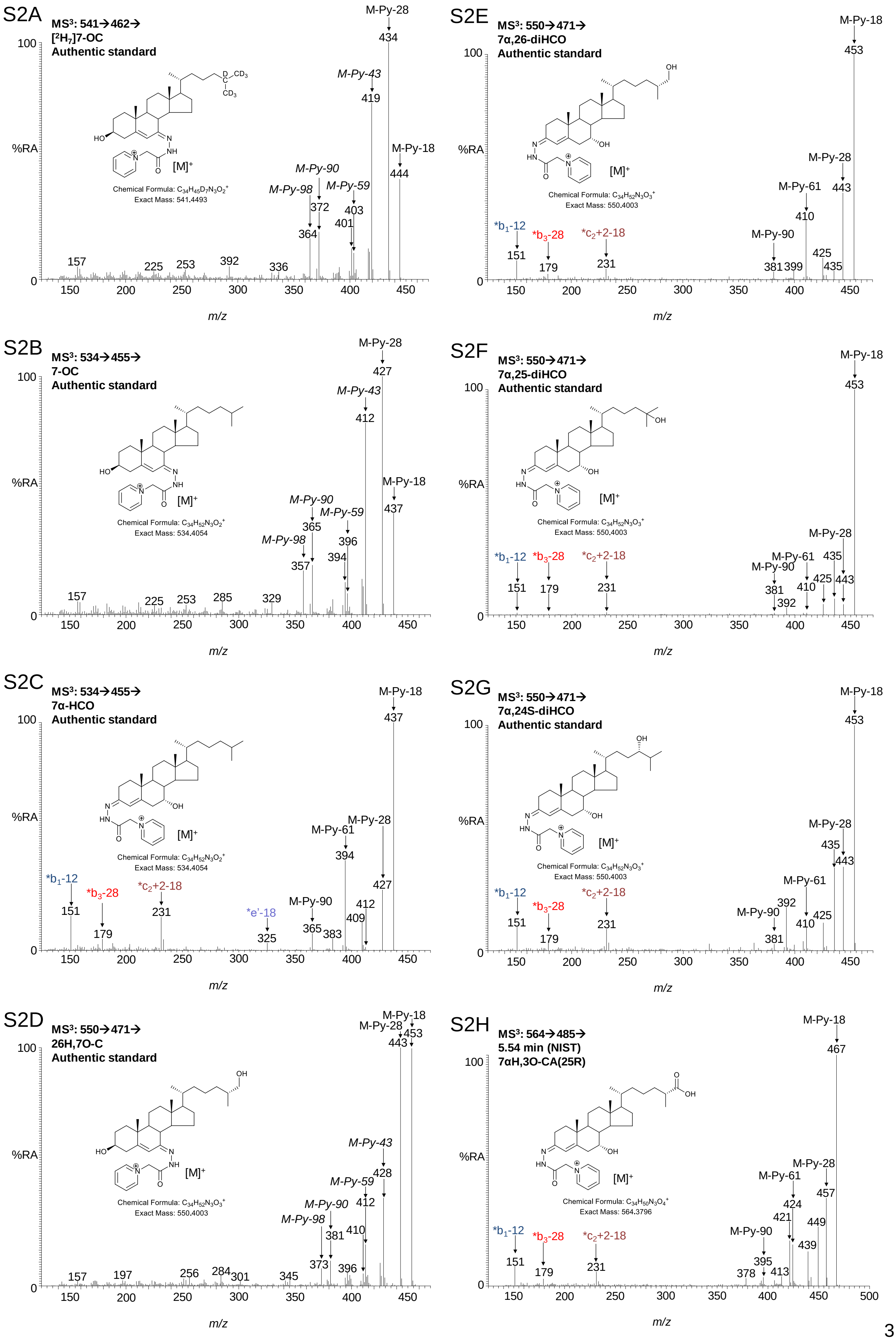

S2I

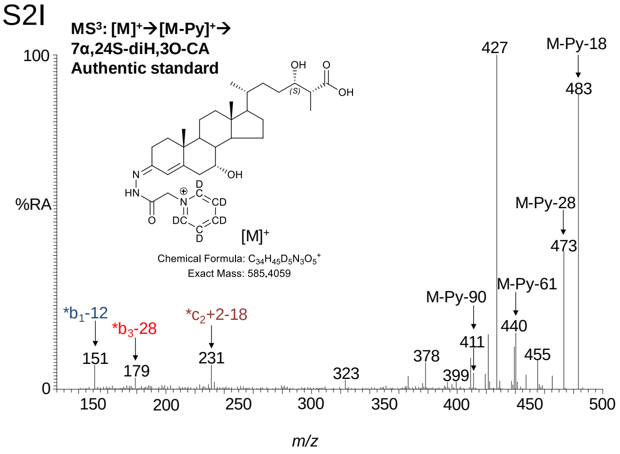

MS³: [M]⁺→[M-Py]⁺→
7α,24S-diH,3O-CA
Authentic standard
[M]⁺
Chemical Formula: C34H45D5N3O5⁺
Exact Mass: 585.4059
100
%RA
0
*b1-12
151
*b3-28
179
*c2+2-18
231
323
378
M-Py-90
411
399
427
M-Py-61
440
455
M-Py-28
473
M-Py-18
483
150
200
250
300
350
400
450
500
m/z

S2J

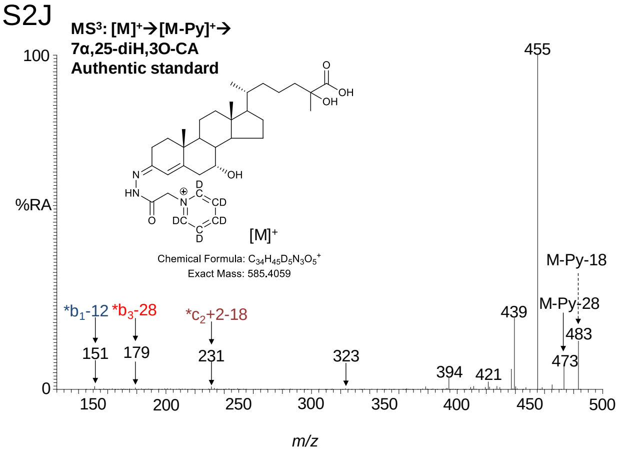

MS³: [M]⁺→[M-Py]⁺→
7α,25-diH,3O-CA
Authentic standard
[M]⁺
Chemical Formula: C34H45D5N3O5⁺
Exact Mass: 585.4059
100
%RA
0
*b1-12
151
*b3-28
179
*c2+2-18
231
323
394
421
439
455
M-Py-18
M-Py-28
483
473
150
200
250
300
350
400
450
500
m/z

S2K

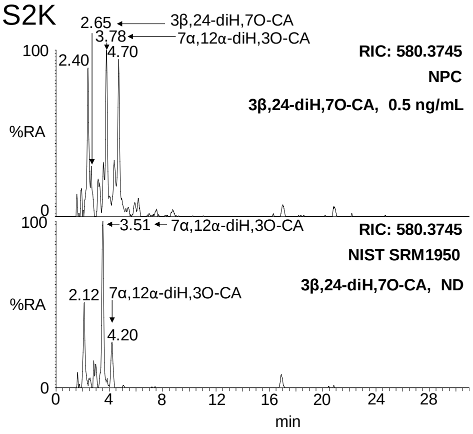

2.65 ← 3β,24-diH,7O-CA
3.78 ← 7α,12α-diH,3O-CA
2.40
4.70
RIC: 580.3745
NPC
3β,24-diH,7O-CA, 0.5 ng/mL
100
%RA
0
← 3.51 ← 7α,12α-diH,3O-CA
RIC: 580.3745
NIST SRM1950
3β,24-diH,7O-CA, ND
2.12
7α,12α-diH,3O-CA
4.20
0
4
8
12
16
20
24
28
min

Supplemental Figure S3

S3A

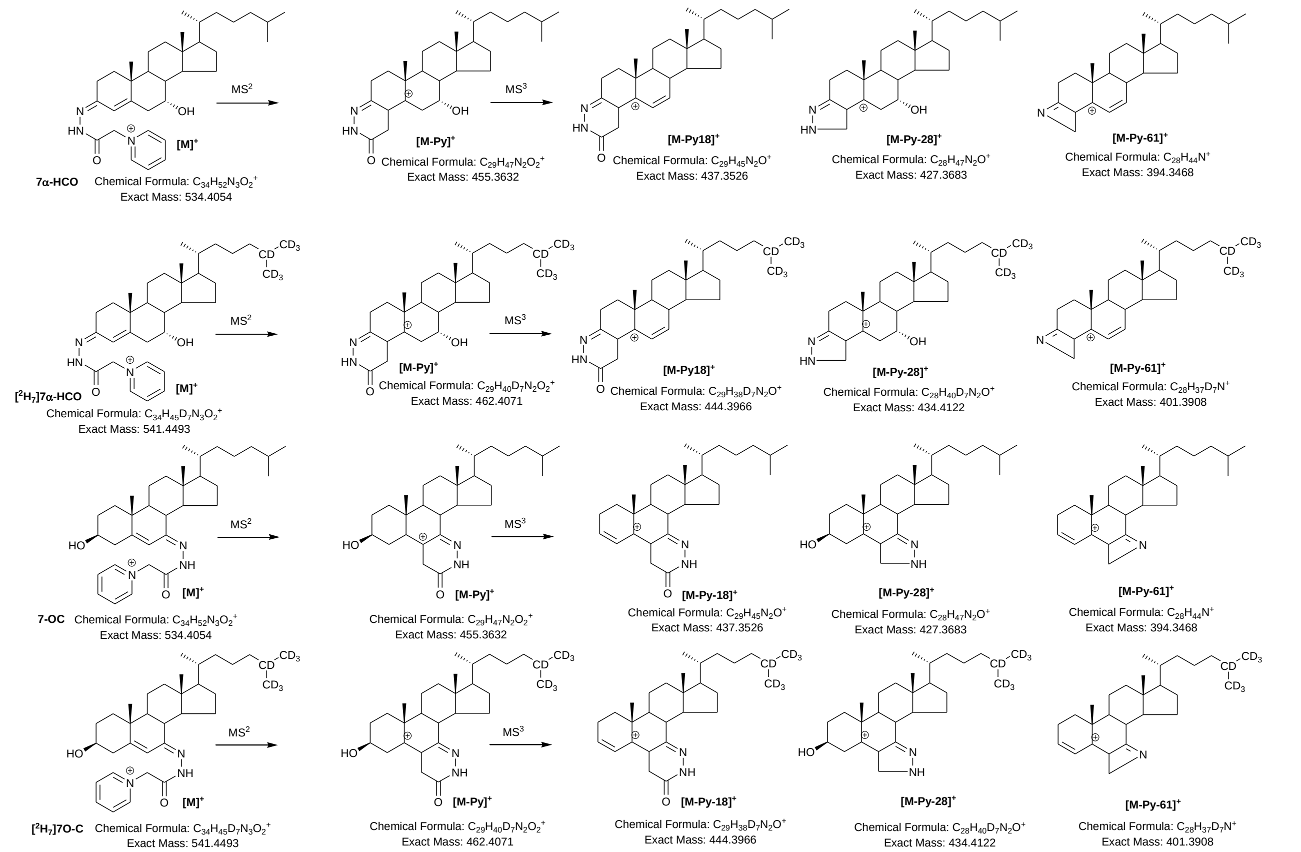

S3B

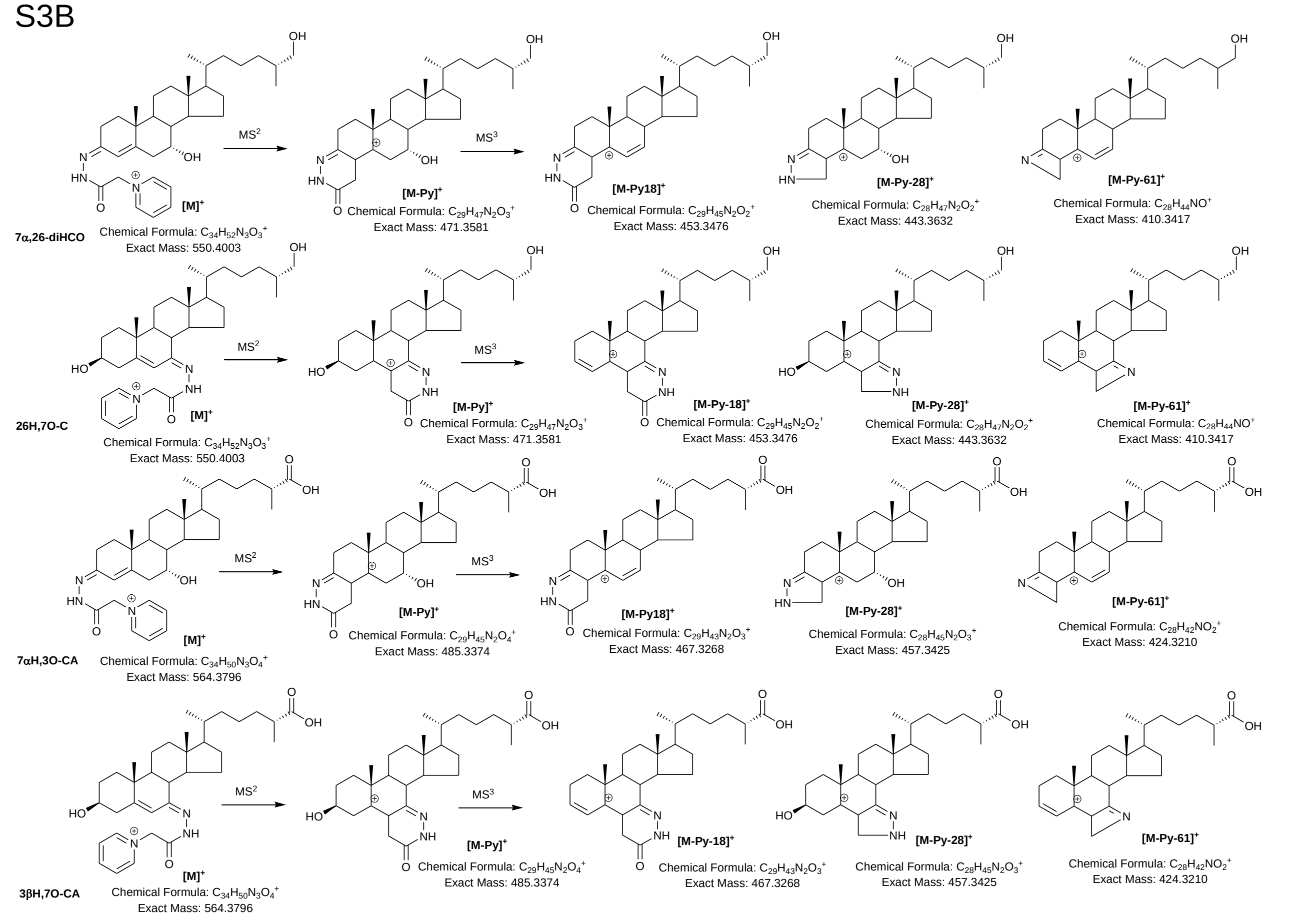

S3C

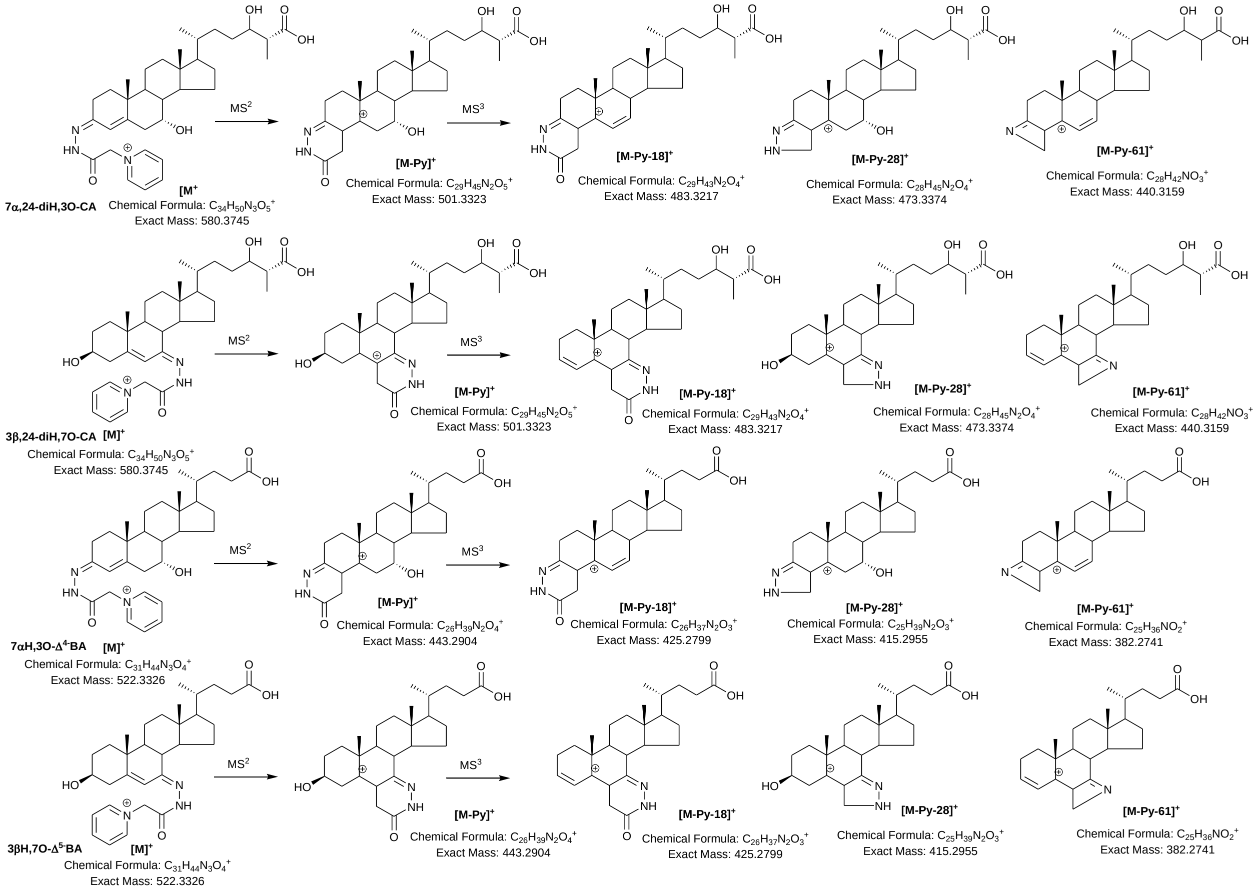
MS^2
MS^3
7α,24-diH,3O-CA
[M$^+$
Chemical Formula: $C_{34}H_{50}N_3O_5^+$
Exact Mass: 580.3745
[M-Py]$^+$
Chemical Formula: $C_{29}H_{45}N_2O_5^+$
Exact Mass: 501.3323
[M-Py-18]$^+$
Chemical Formula: $C_{29}H_{43}N_2O_4^+$
Exact Mass: 483.3217
[M-Py-28]$^+$
Chemical Formula: $C_{28}H_{45}N_2O_4^+$
Exact Mass: 473.3374
[M-Py-61]$^+$
Chemical Formula: $C_{28}H_{42}NO_3^+$
Exact Mass: 440.3159
3β,24-diH,7O-CA
[M]$^+$
Chemical Formula: $C_{34}H_{50}N_3O_5^+$
Exact Mass: 580.3745
[M-Py]$^+$
Chemical Formula: $C_{29}H_{45}N_2O_5^+$
Exact Mass: 501.3323
[M-Py-18]$^+$
Chemical Formula: $C_{29}H_{43}N_2O_4^+$
Exact Mass: 483.3217
[M-Py-28]$^+$
Chemical Formula: $C_{28}H_{45}N_2O_4^+$
Exact Mass: 473.3374
[M-Py-61]$^+$
Chemical Formula: $C_{28}H_{42}NO_3^+$
Exact Mass: 440.3159
7αH,3O-Δ^4-BA
[M]$^+$
Chemical Formula: $C_{31}H_{44}N_3O_4^+$
Exact Mass: 522.3326
[M-Py]$^+$
Chemical Formula: $C_{26}H_{39}N_2O_4^+$
Exact Mass: 443.2904
[M-Py-18]$^+$
Chemical Formula: $C_{26}H_{37}N_2O_3^+$
Exact Mass: 425.2799
[M-Py-28]$^+$
Chemical Formula: $C_{25}H_{39}N_2O_3^+$
Exact Mass: 415.2955
[M-Py-61]$^+$
Chemical Formula: $C_{25}H_{36}NO_2^+$
Exact Mass: 382.2741
3βH,7O-Δ^5-BA
[M]$^+$
Chemical Formula: $C_{31}H_{44}N_3O_4^+$
Exact Mass: 522.3326
[M-Py]$^+$
Chemical Formula: $C_{26}H_{39}N_2O_4^+$
Exact Mass: 443.2904
[M-Py-18]$^+$
Chemical Formula: $C_{26}H_{37}N_2O_3^+$
Exact Mass: 425.2799
[M-Py-28]$^+$
Chemical Formula: $C_{25}H_{39}N_2O_3^+$
Exact Mass: 415.2955
[M-Py-61]$^+$
Chemical Formula: $C_{25}H_{36}NO_2^+$
Exact Mass: 382.2741

Supplemental Figure S4

S4A

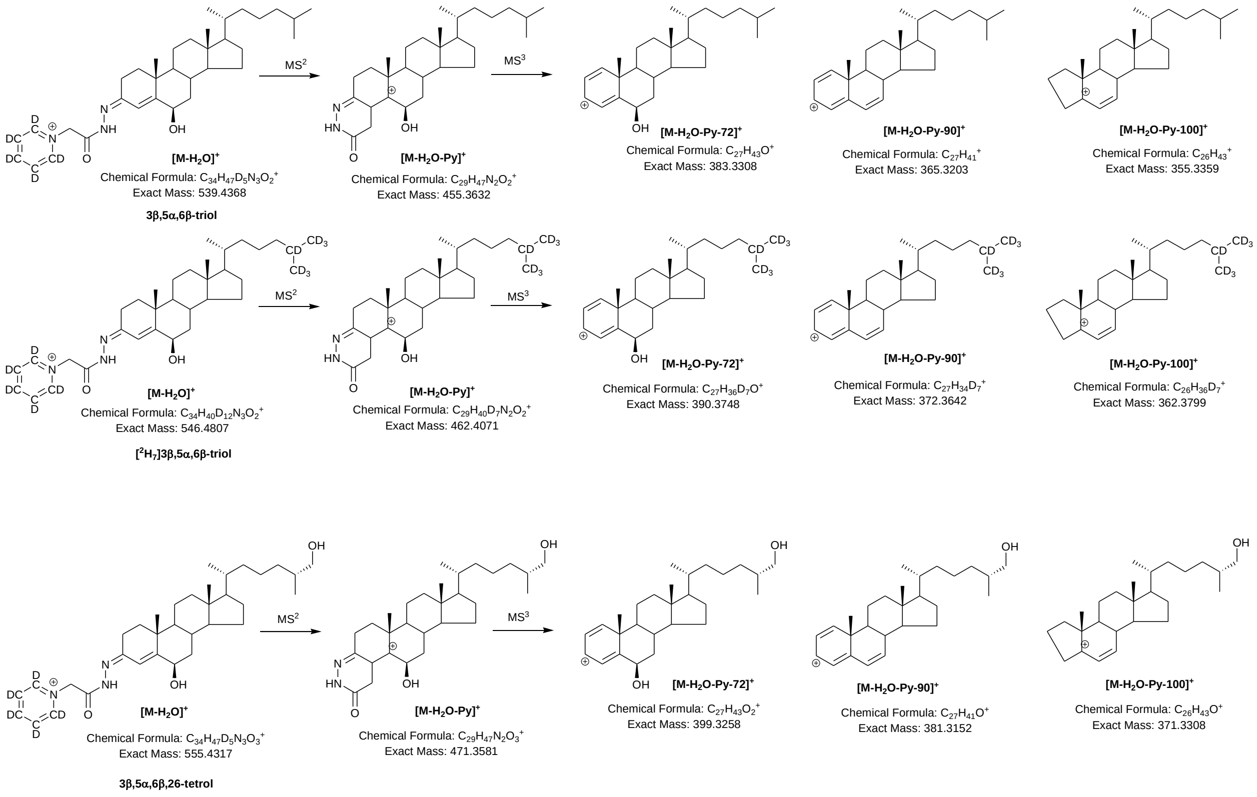

S4B

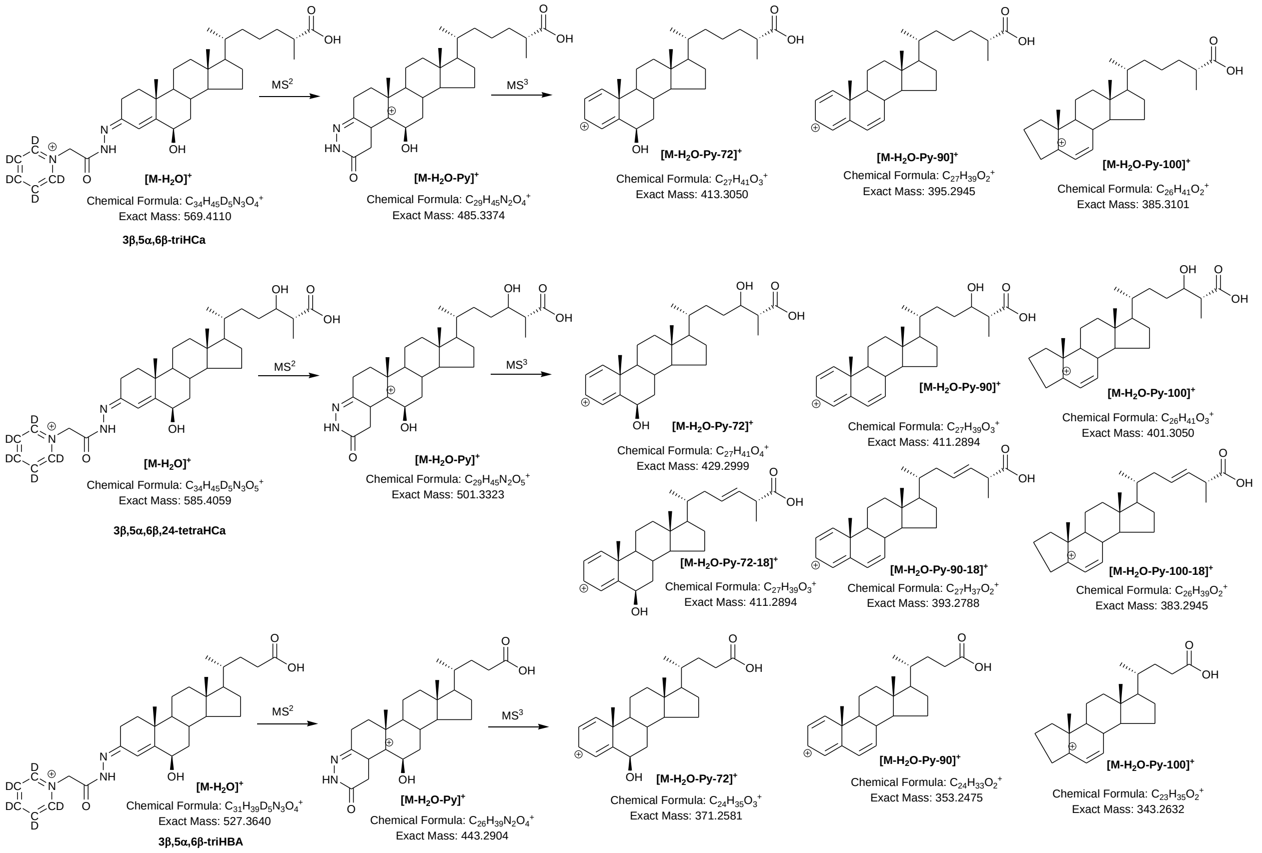

Supplemental Figure S5

S5A

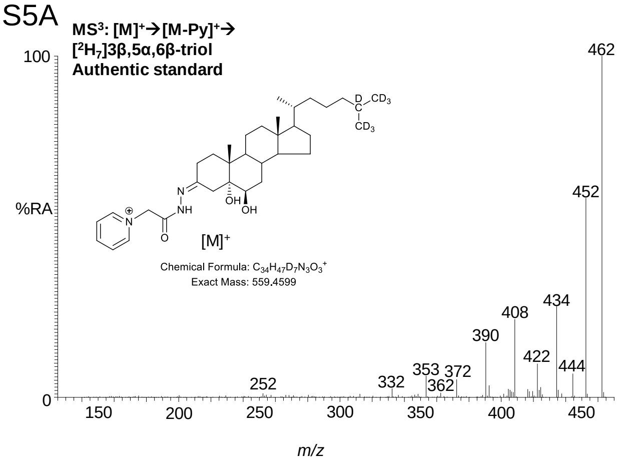

S5E

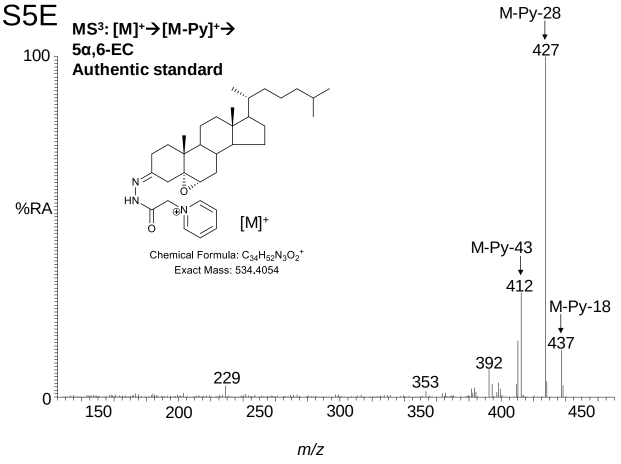

S5B

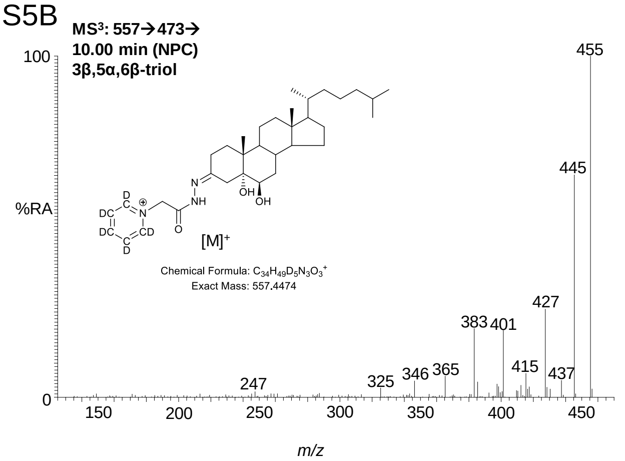

S5F

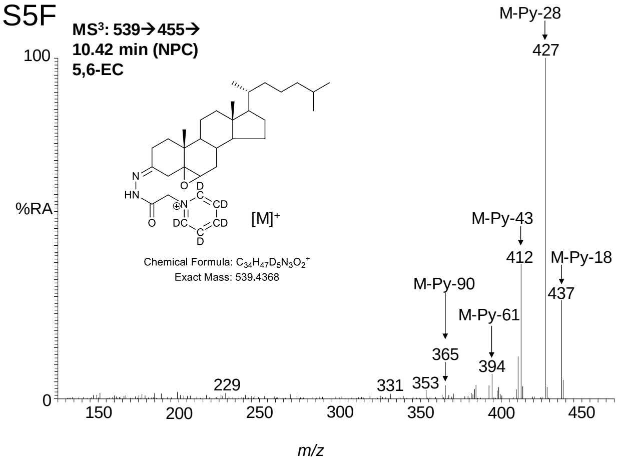

S5C

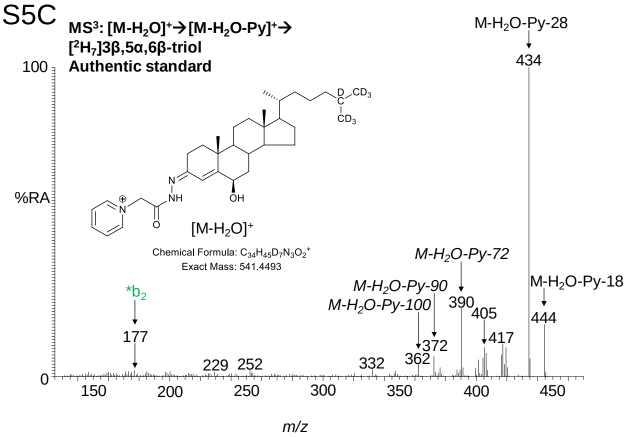

S5G

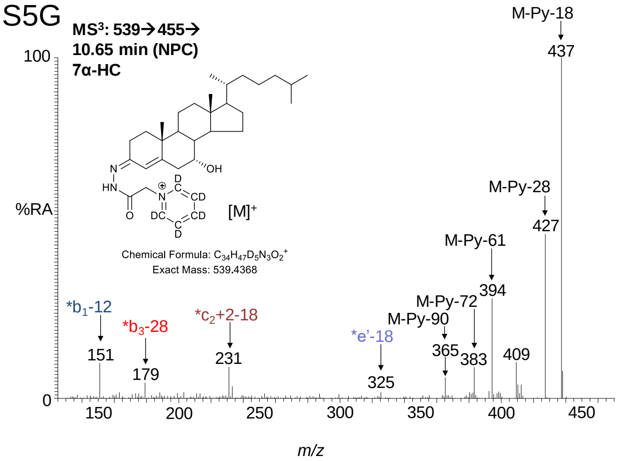

S5D

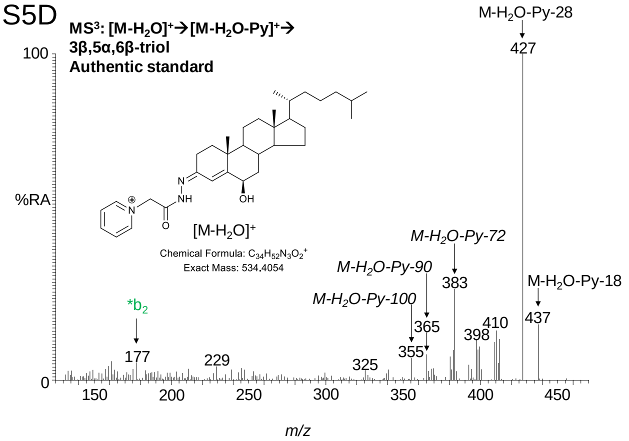

S5H

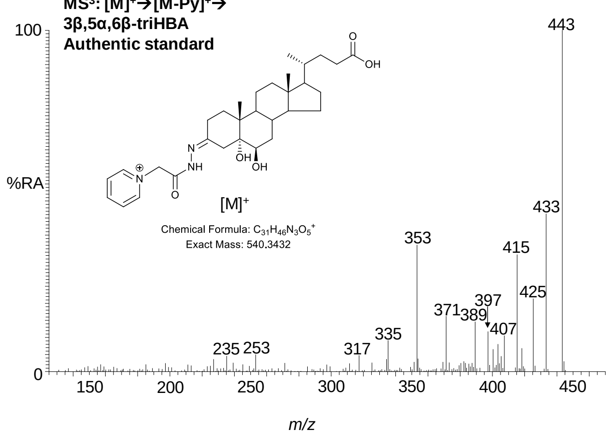

S5I

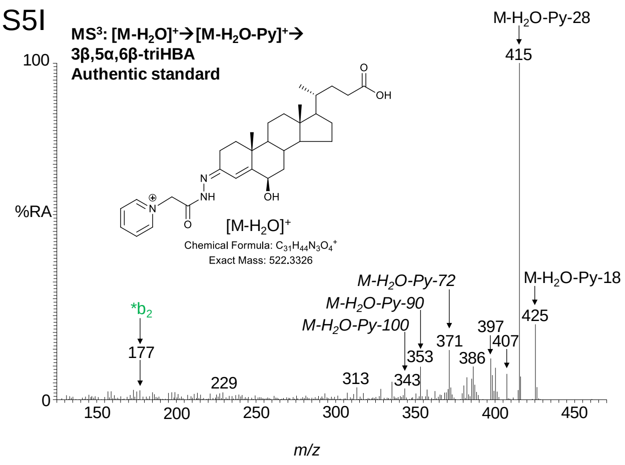

S5M

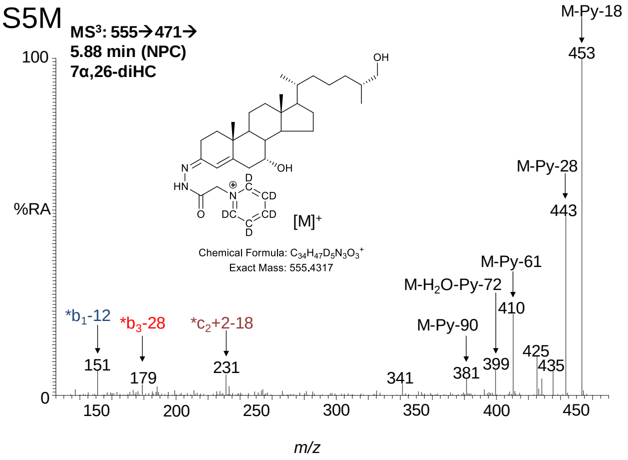

S5J

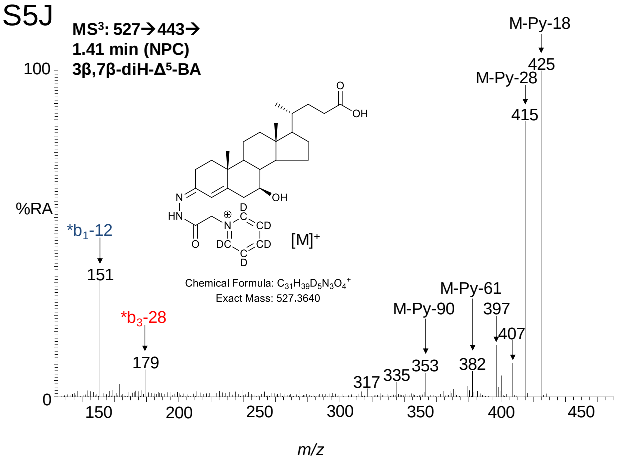

S5N

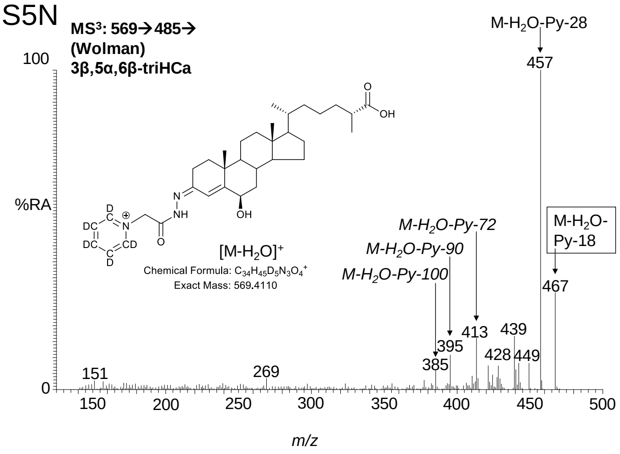

S5K

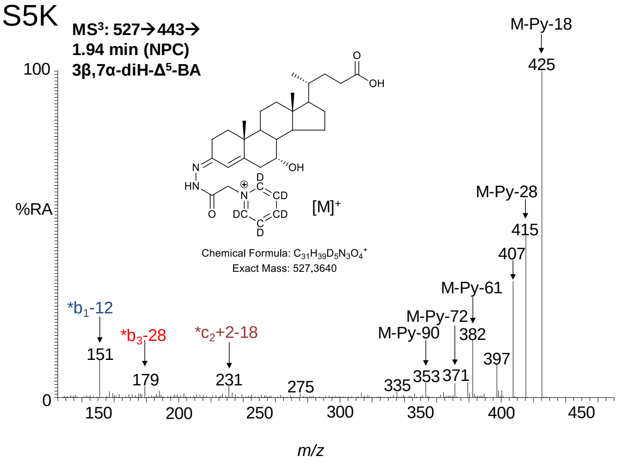

S5O

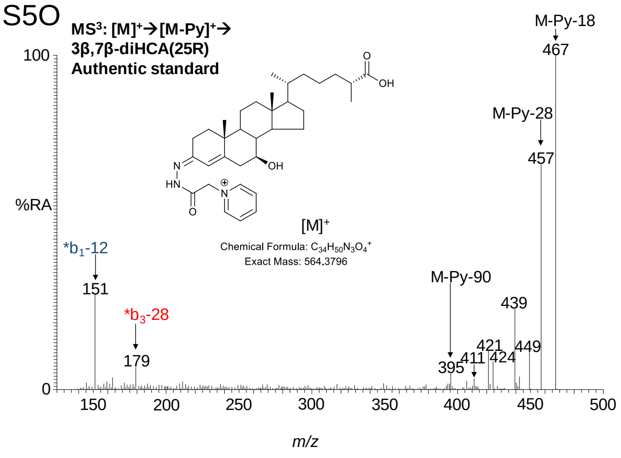

S5L

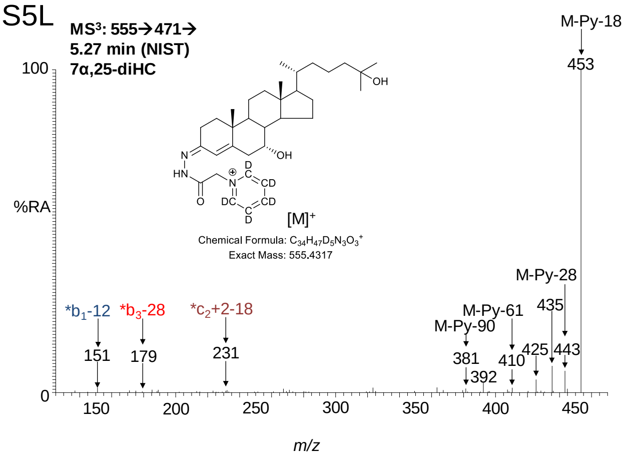

S5P

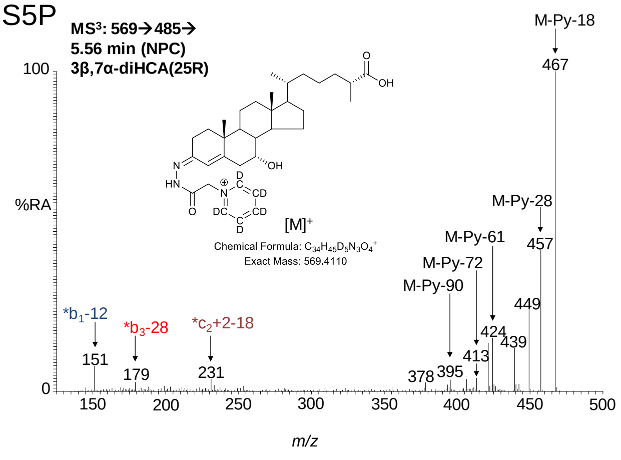

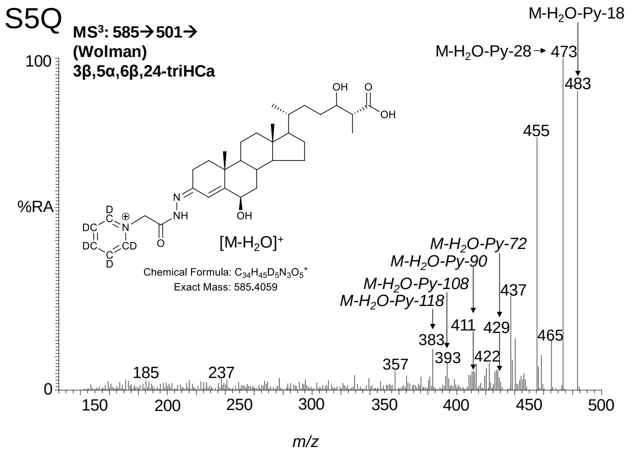
S5Q
MS³: 585→501→
(Wolman)
3β,5α,6β,24-triHCa
M-H2O-Py-18
M-H2O-Py-28
473
483
455
[M-H2O]+
Chemical Formula: C34H45D5N3O5+
Exact Mass: 585.4059
M-H2O-Py-72
M-H2O-Py-90
M-H2O-Py-108
M-H2O-Py-118
437
411
429
383
465
393
422
357
185
237
%RA
100
0
150
200
250
300
350
400
450
500
m/z

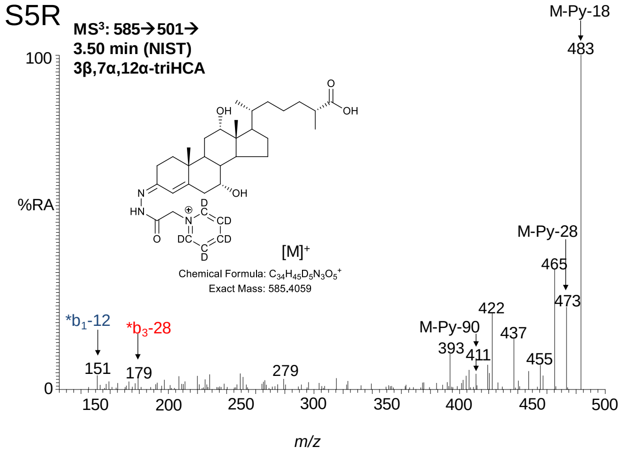
S5R
MS³: 585→501→
3.50 min (NIST)
3β,7α,12α-triHCA
M-Py-18
483
[M]+
Chemical Formula: C34H45D5N3O5+
Exact Mass: 585.4059
M-Py-28
465
473
422
M-Py-90
437
393
411
455
*b1-12
*b3-28
151
179
279
%RA
100
0
150
200
250
300
350
400
450
500
m/z
